# A transition metal-free route to *N*-functionalised ester substituted isoquinolinones

**DOI:** 10.1039/d6ra05642c

**Published:** 2026-07-03

**Authors:** Hannah Chapman, Callum Dutton, Helen F. Sneddon

**Affiliations:** a Green Chemistry Centre of Excellence, Department of Chemistry, University of York Heslington York YO10 5DD UK helen.sneddon@york.ac.uk

## Abstract

Isoquinolinones are important heterocycles across a range of natural products, pharmaceuticals and agrochemicals. Attempts to conduct an S_N_2 displacement of the bromide of methyl (*E*)-2-(2-(bromomethyl)phenyl)-3-methoxyacrylate with an amine proceeded with concomitant cyclisation and oxidation to generate an ester substituted isoquinolinone. This approach represents a new, transition metal-free approach for isoquinolinone synthesis and the scope and limitations are explored.

## Abstract 2

A mild, transition-metal free cyclisation forming isoquinolinones has been demonstrated for a range of amines in a range of solvents.

## Graphical abstract

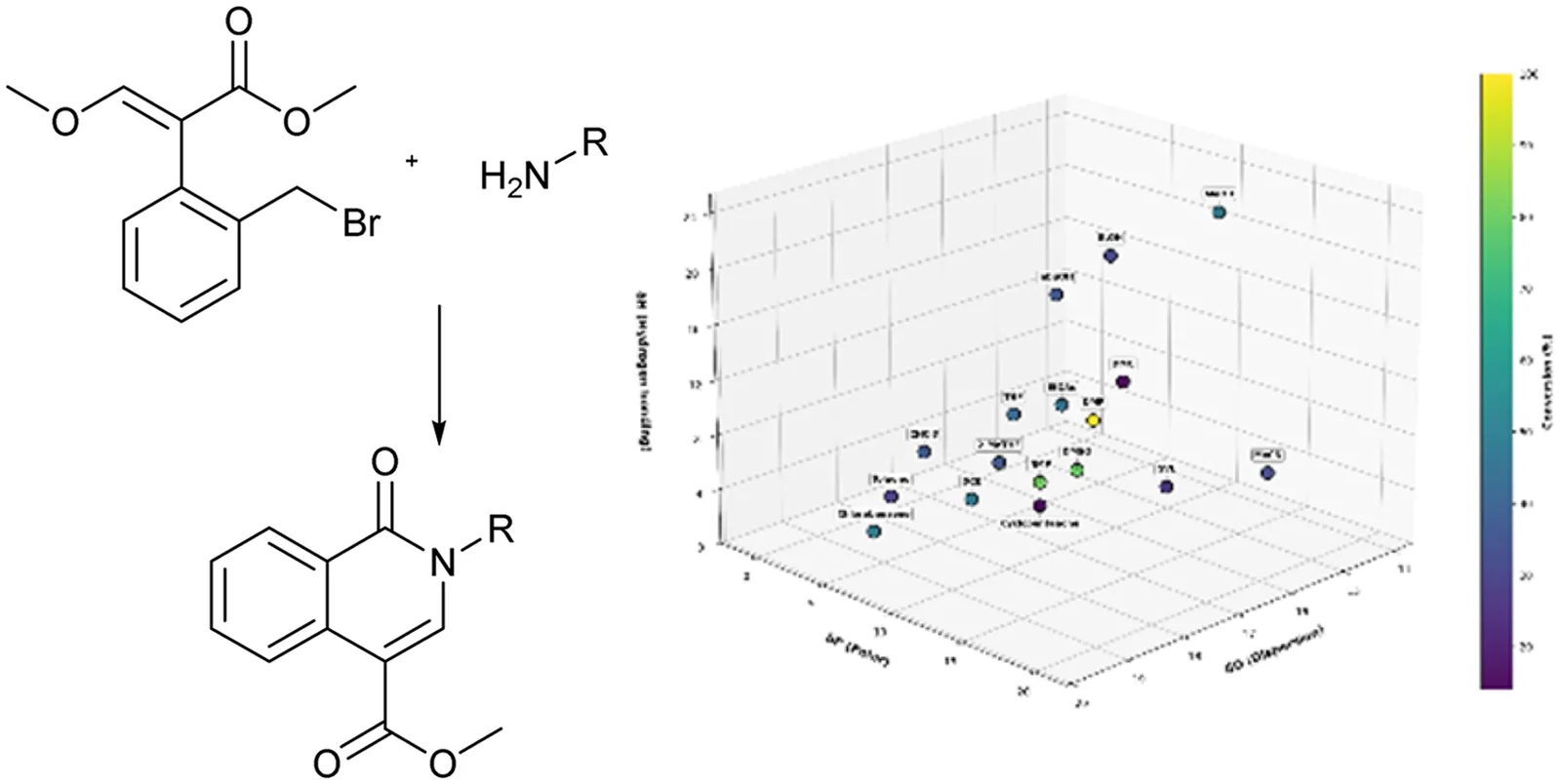

## Introduction

**Fig. 1:**
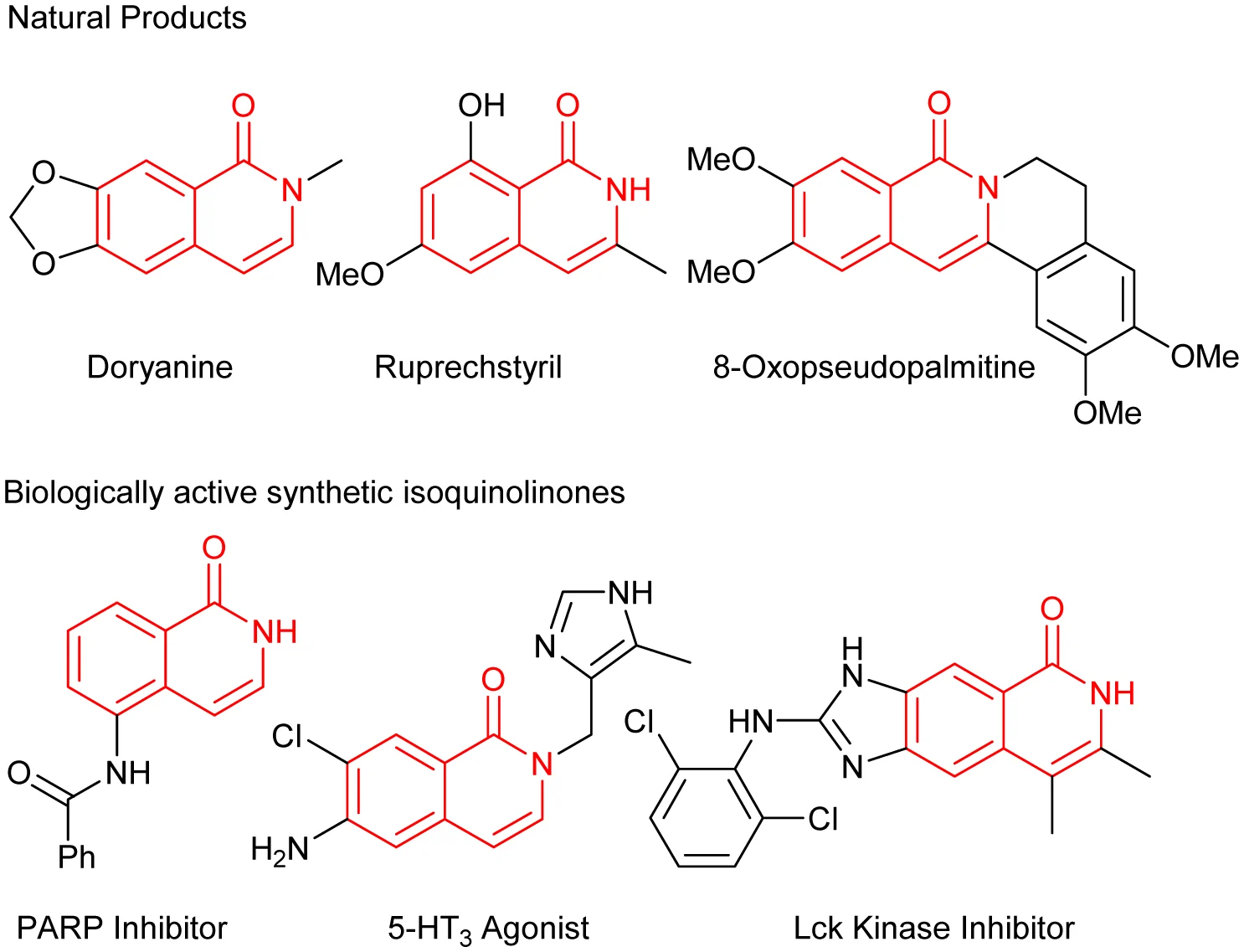
Selected isoquinolinone containing natural products and compounds of biological interest.

**Fig. 2:**
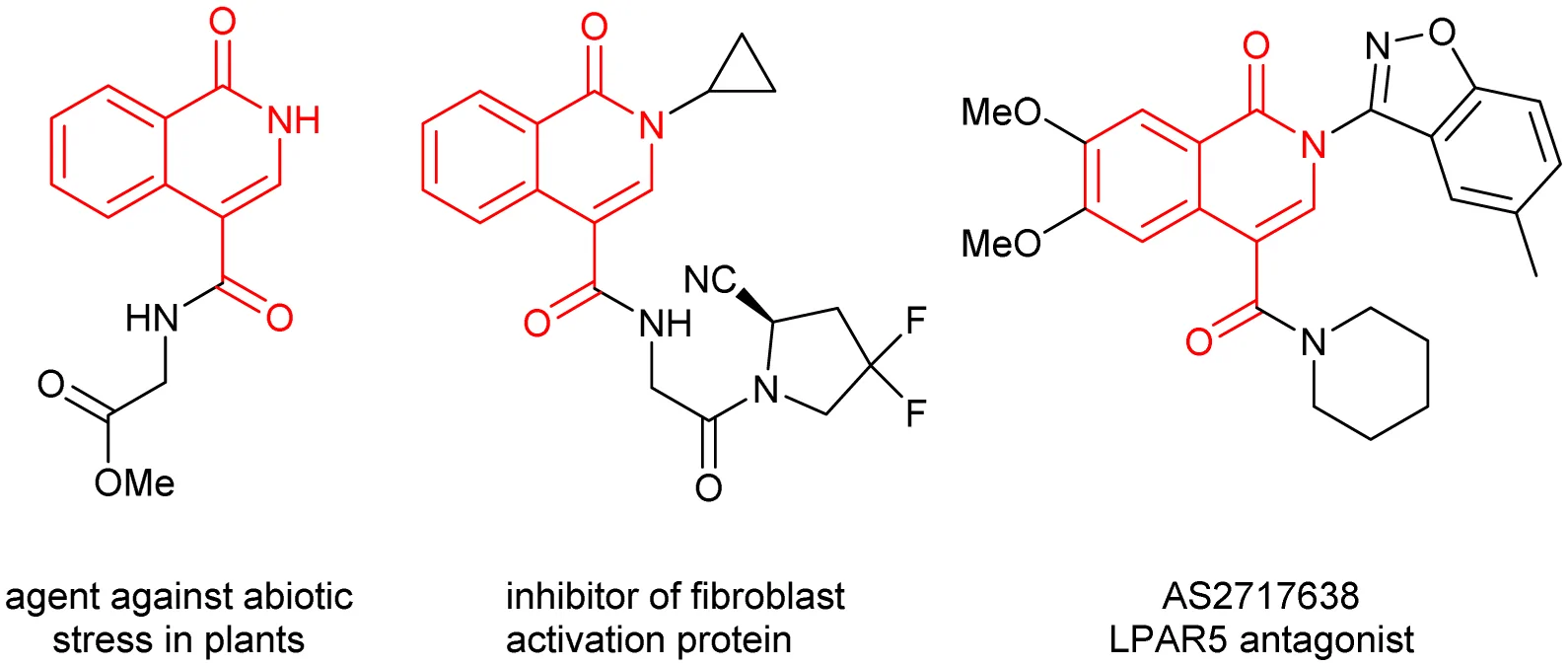
Selected 4-functionalised isoquinolinones of biological interest.

Isoquinolinones are heterocycles of particular interest in biologically active natural products, such as doryanine,^1^ ruprechstyril,^2^ 8-oxopseudopalmitine,^3^ amongst others.^4^ They are also of importance in drug development where they feature as a scaffold across a wide range of therapeutic areas, including as PARP inhibitors,^5^ 5-HT_3_ agonists,^6^ rho kinase inhibitors,^7^ anti-hypertension agents,^8^ anti-diabetic agents,^9^ anti-inflammation agents^10^ and antimalarials;^3^ and in agrochemicals where they are found for example in fungicides^11^ (Fig. 1). More specifically isoquinolinones with carbonyls at the 4 position have been listed as compounds of interest as active agents against abiotic stress in plants,^12^ and as inhibitors of fibroblast activation protein,^13^ and as LPAR5 antagonists^14^ amongst other applications (Fig. 2).

**Fig. 3:**
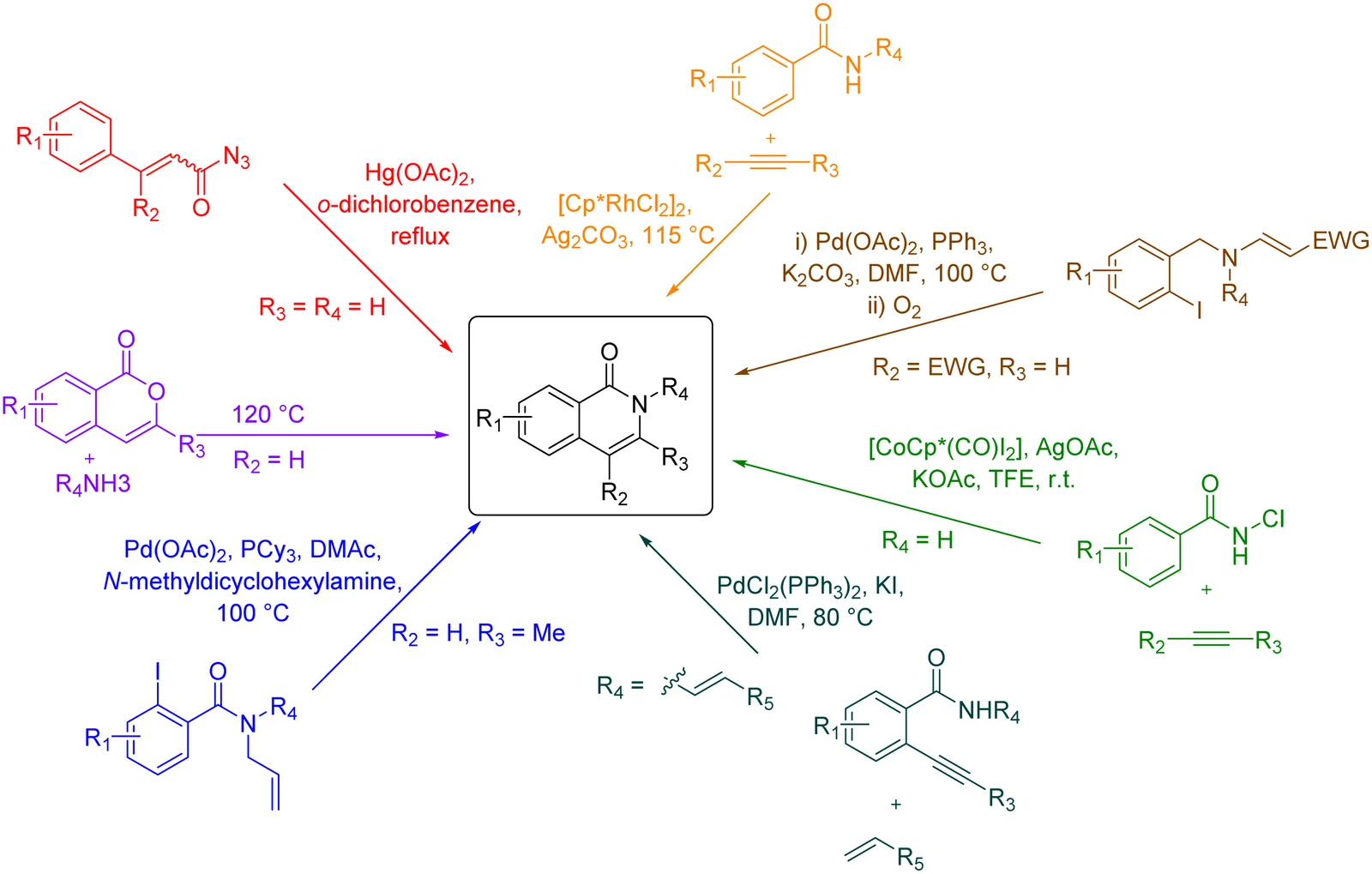
Selected synthetic routes to isoquinolinones.

Numerous approaches to isoquinolinone synthesis have been developed (Fig. 3). These have included classical methods such as modifications of the Bischler–Napieralski^15^ and Pictet–Spengler^16^ reactions, however they generally required pre-modified reagents, extreme conditions, and suffered from a narrow substrate scope. Isoquinolinone syntheses by annulation methods – exploring Ni/Rh/Ru and Co systems have recently been comprehensively reviewed by Hua,^17^ and catalytic approaches, including electrocatalysis, photocatalysis, transition metal catalysed and metal-free approaches have been recently reviewed by Kumar.^18^ Guimond and Fagnou disclosed a Rh catalysed synthesis of isoquinolones through an oxidative coupling of benzamides and alkynes.^19^ Chuang and Wu^20^ developed Hg(OAc)_2_ catalysed Curtius reactions and intramolecular cyclisations of cinnamoyl azides. In addition, many research groups explored the synthesis of isoquinolinone structures through Heck reactions of pre-functionalised compounds. Pre-functionalisation has been explored through both the Ugi reaction (by Dömling *et al.*)^21^ and through forming 2-alkynyl-benzamides from a Sonogashira reaction of 2-halo-benzamides (by Yao *et al*).^22^ De Mesmaeker in 1998, when exploring solid supported synthesis, coupled Heck reactions with oxygen oxidation to form isoquinolinones.^23^ Isoquinolinones can also be formed by recyclization of isocoumarins/isochromenones.^24^

**Fig. 4:**
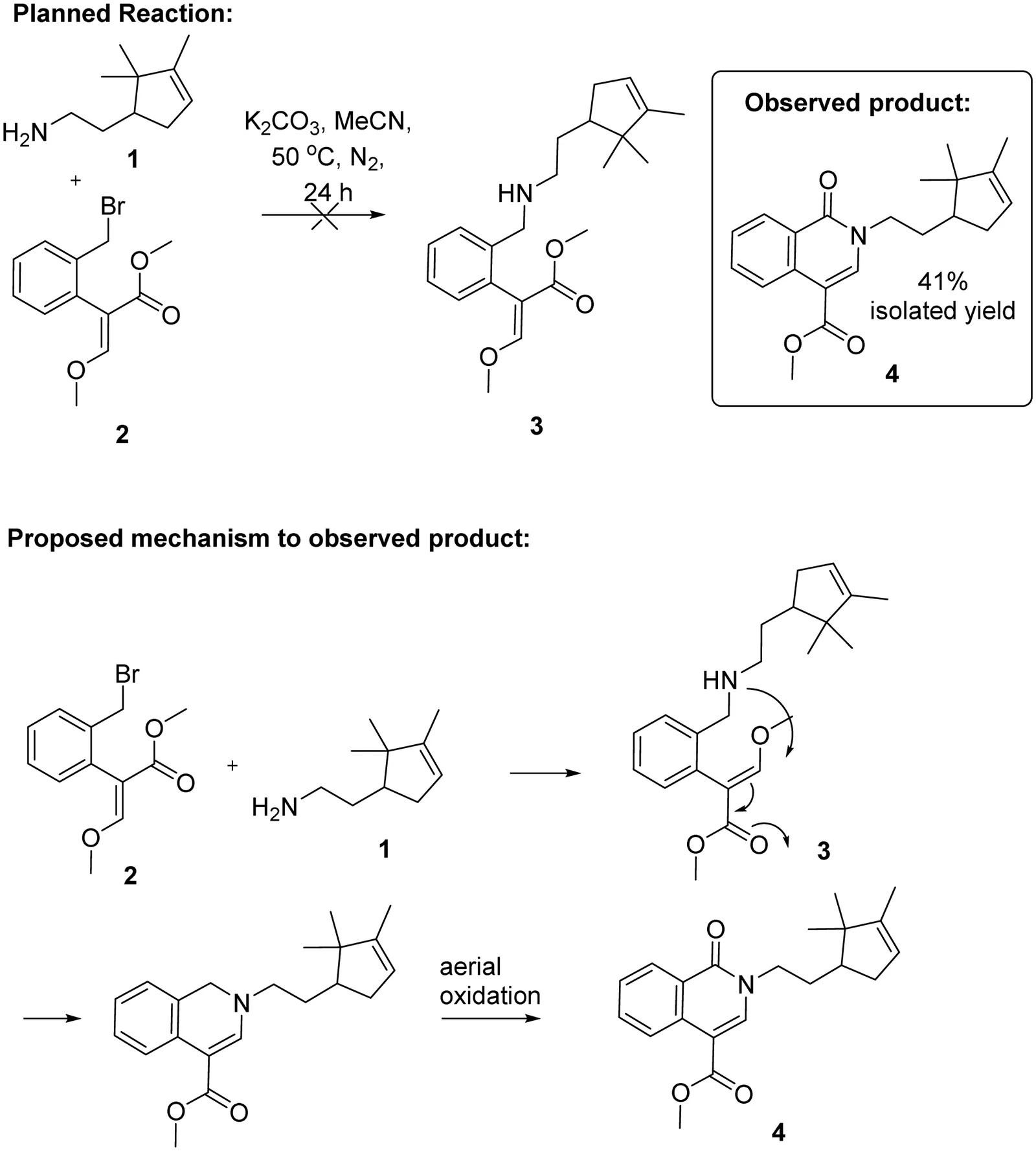
Serendipitous discovery of an air-mediated isoquinolinone synthesis.

In a separate project, we were interested in accessing the benzylamine (3) by displacement of the bromide from methyl (*E*)-2-(2-(bromomethyl)phenyl)-3-methoxyacrylate (2)^25–28^ with the α-pinene derived 2-(2,2,3-trimethylcyclopent-3-en-1-yl)ethan-1-amine (1).^29^ To our surprise, only isoquinolinone (4) was isolated. The formation of this product could be rationalised by the secondary amine formed on S_N_2 displacement of the bromide being able to perform a conjugate addition onto the α,β-unsaturated ester, followed by elimination of methanol. Presumably, the dihydroisoquinoline which results is then oxidised in air to form the isoquinolinone, a transformation previously explored by Deng *et al.*^30^ (Fig. 4).

We realised that this unexpected reaction could be a useful way of synthesising substituted isoquinolinones. Thus, herein, we now report a study on the scope and limitations of this transition metal-free route to a heterocycle motif with useful biological properties.

## Results and discussion

**Scheme 1:**
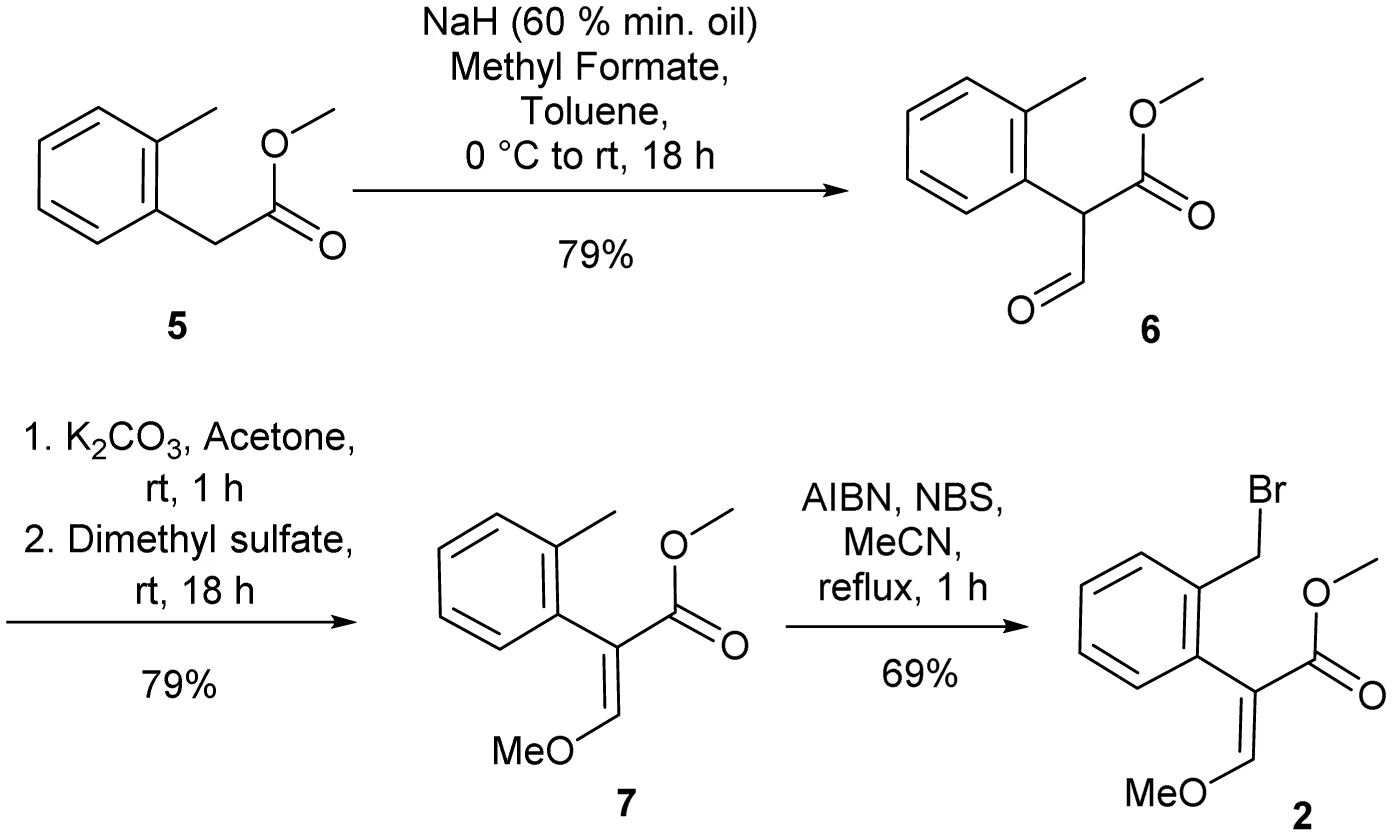
Synthesis of methyl (*E*)-2-(2-(bromomethyl)phenyl)-3-methoxyacrylate.

The starting point was the synthesis of methyl (*E*)-2-(2-(bromomethyl)phenyl)-3-methoxyacrylate (2), reproducing steps which have previously been reported.^25–28^ Commercially-available methyl 2-(*o*-tolyl)acetate (5) was deprotonated with sodium hydride and condensed with methyl formate to yield methyl 3-oxo-2-(*o*-tolyl)propanoate (6)^31^ in 79% yield. Methylation with dimethyl sulfate afforded a separable mixture of *E* and *Z* methyl-3-methoxy-2-(*o*-tolyl) acrylate (7),^31^ and bromination with NBS afforded the desired benzyl bromide (2)^28^ (Scheme 1).

Attempts were made to isolate the purported dihydroisoquinolinone 8. Whilst this species could clearly be seen by mass spectrometry, by ^1^H NMR spectroscopy and observed by GC (see SI), this compound could not be isolated in our studies in high purity, as it oxidised in air to the isoquinolinone, as is consistent with our proposed mechanism. Whilst the initial reaction was conducted under a balloon of nitrogen, we hypothesise that oxidation could be occurring courtesy of the oxygen present in the solvent, on exposure to air during isolation, or in less rigorously conducted reactions through the atmosphere above the solvent.

A brief exploration of the reaction conditions and scope of the cyclisation and oxidative approach to isoquinolinones from methyl (*E*)-2-(2-(bromomethyl)phenyl)-3-methoxyacrylate (2) was undertaken. Mindful of photochemically mediated oxidations of tetrahydroisoquinolines to isoquinolinones reported in the literature,^32^ and the proposed involvement of iodine reported by Deng and co-workers,^30^ the effect of light and iodine, were explored, in addition to a small range of bases, with conversions measured by GC (Table 1).

**Table 1:** Screening of conditions^a^

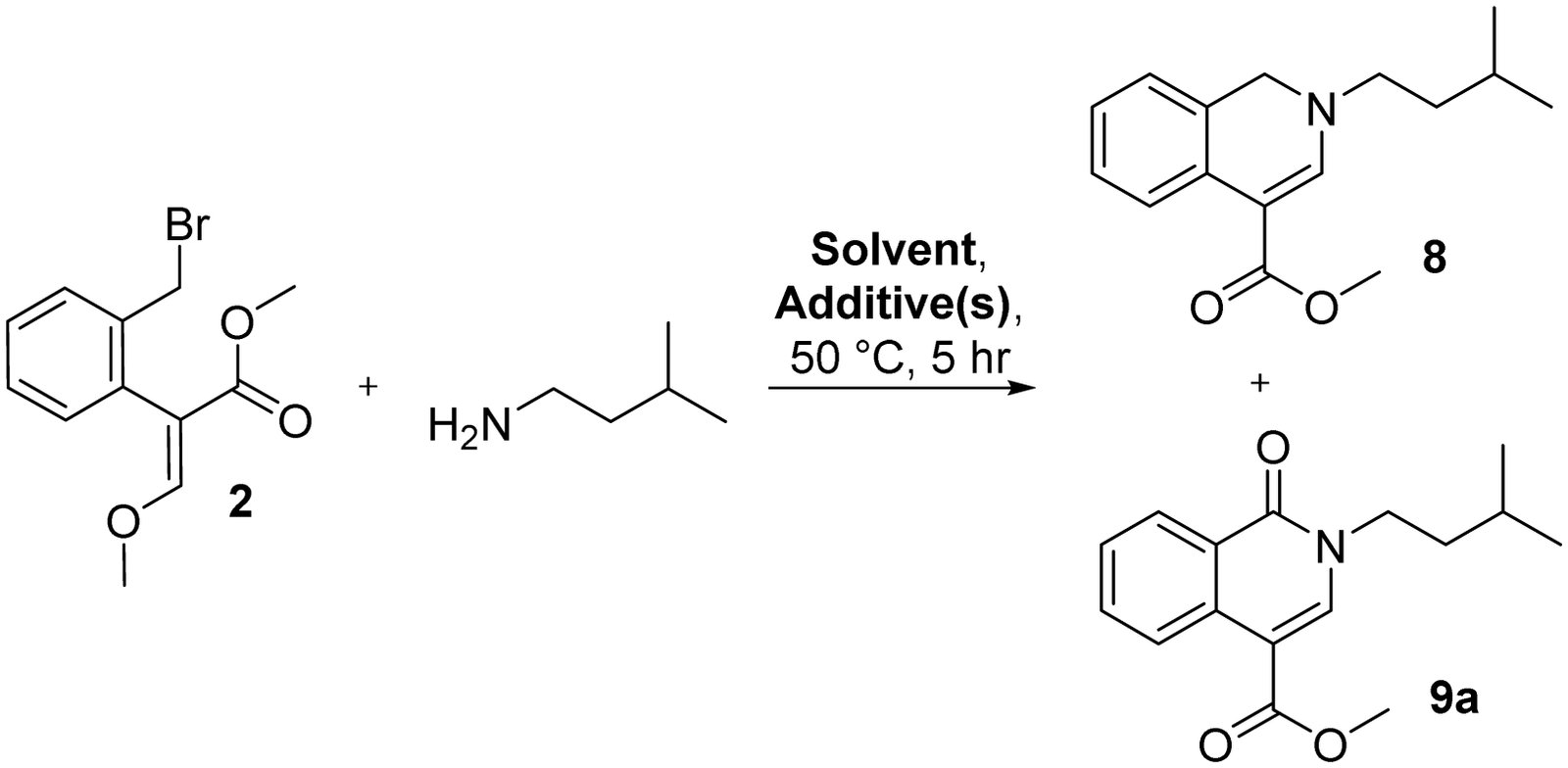		
Solvent	Additive(s)	Conversion after 5 h (%)
MeCN	None	34
MeCN	0.1 eq. Barton's base	17
MeCN	0.1 eq. DMAP	33
MeCN	1.3 eq. K_2_CO_3_	23
MeCN	1.3 eq. DMAP	3
MeCN	0.1 eq. DMAP	33
MeCN	0.1 eq. DMAP, foil wrapped (dark)	32
MeCN	0.1 eq. DMAP, 0.15 eq. I_2_	22
EtOAc	0.1 eq. DMAP, 0.15 eq. I_2_ after 3 h	17

a GC conversion.

Screening of a handful of different bases, comparing the conversions to product by GC, and the ratio of cyclised intermediate to oxidised final product by ^1^H NMR spectroscopy of the crude product, suggested that whilst base promoted the initial cyclisation, it inhibited the subsequent oxidation. Whilst iodine might be proposed to assist in the oxidation of tetrahydroisoquinolines to isoquinolinones,^30^ in our hands for these reactions, conversion to the desired product was better in the absence of iodine, and the presence or absence of light did not appear significant.

**Fig. 5:**
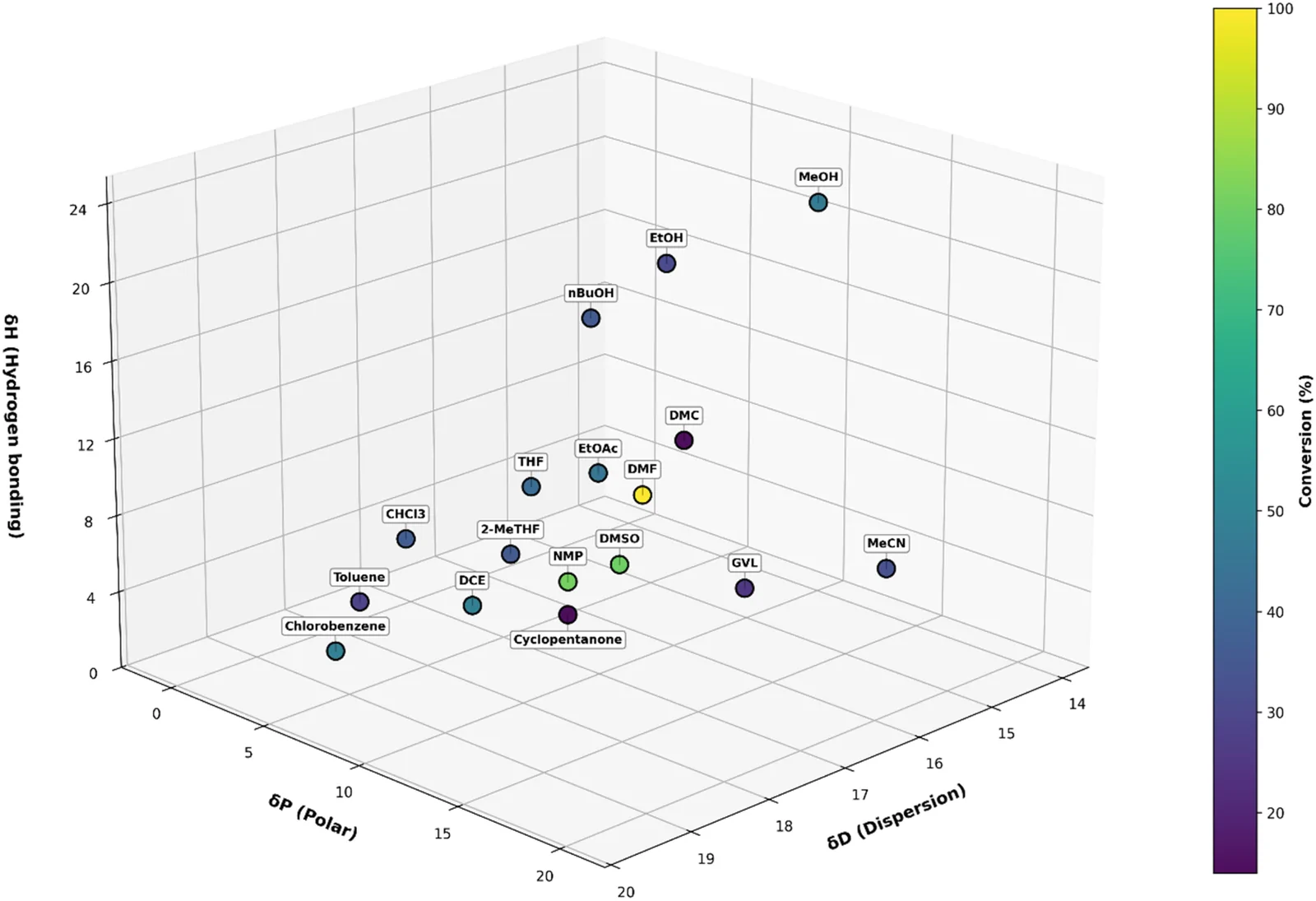
Initial exploration of solvent on cyclisation and oxidation of methyl (*E*)-2-(2-(bromomethyl)phenyl)-3-methoxyacrylate and amines to form isoquinolinones.

Further to the initial results observed in acetonitrile, solvents covering a wide range of Hansen solubility space^33^ were screened, with the conversion to isoquinolinone being measured *via* GC (Fig. 5, full data in SI). DMF, DMSO and NMP gave the most promising yields, and unsurprisingly gamma valerolactone and cyclopentanone appeared to be incompatible with amines, giving lower conversions.

**Fig. 6:**
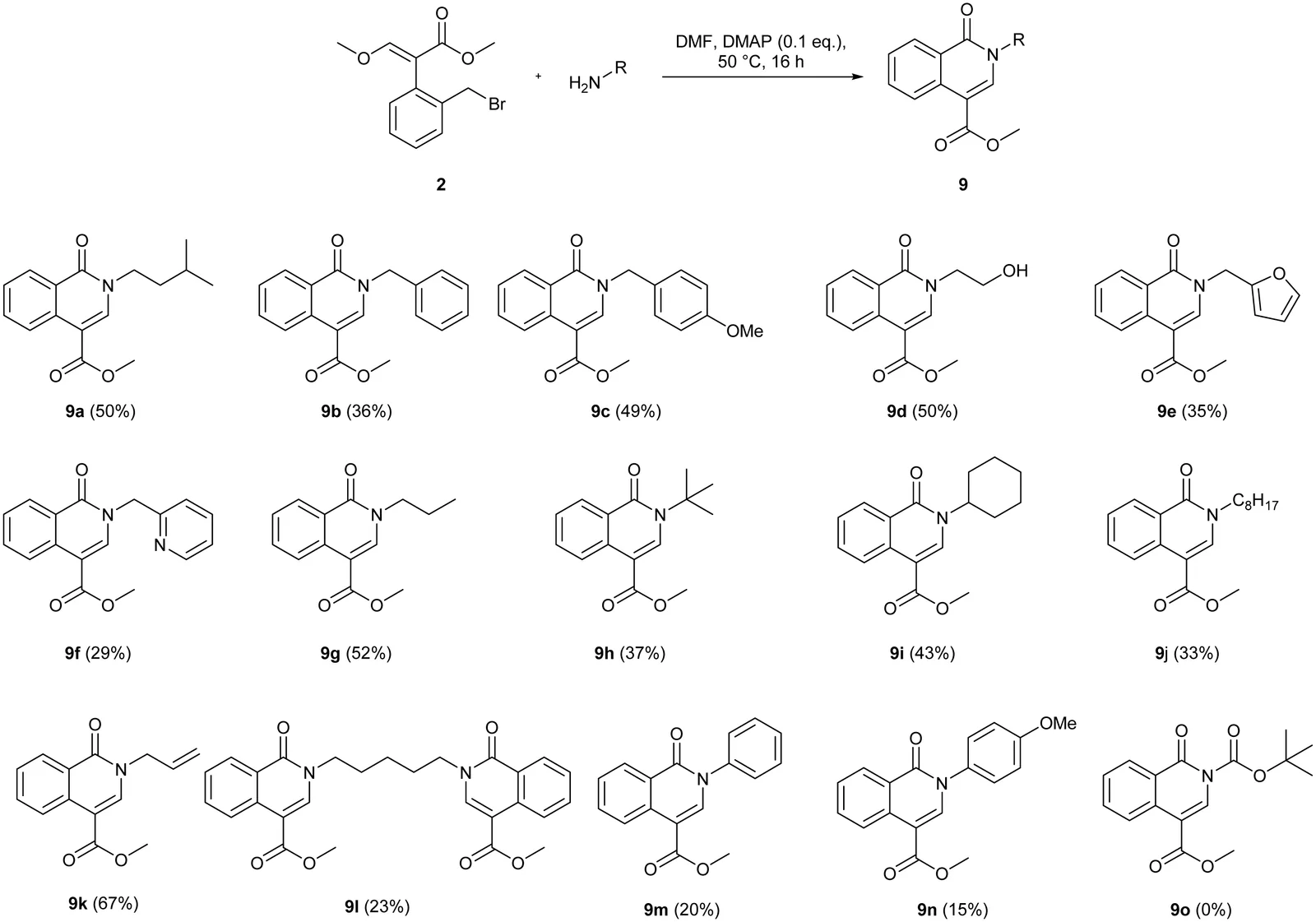
Exploration of amine scope in cyclisation and oxidation of methyl (*E*)-2-(2-(bromomethyl)phenyl)-3-methoxyacrylate and amines to form isoquinolinones (isolated yields).

Mindful of the reprotoxicity and other sustainability concerns of DMF^34^ attempts were made to explore “greener” solvents, in particular through exploring solvent mixtures with similar Hansen solubility parameters to DMF.^35^ However, DMF gave the highest conversions by GC (Table 2), and so this solvent was used for the exploration of scope of the reaction (Fig. 6).

**Table 2:** Exploration of solvent mixtures mimicking the Hansen solubility parameters of DMF

Solvent	dD	dP	dH	Conversion after 5 h (%)
DMF	17.40	13.70	11.30	100
60% THF, 40% glycerol carbonate	17.24	13.62	11.76	17
70% Sulfolane, 30% isopentyl alcohol	17.20	13.74	10.08	58
70% DMSO, 30% pentan-1-ol	17.65	13.25	11.31	71

The scope of the reaction was explored with a range of amines, and low to moderate yields were achieved after 16 hours with aliphatic (9a, 9d, 9g–9l) and non-sterically hindered benzylic primary amines (9b, 9c, 9e, 9f) typically giving the highest yields. The modest yields observed for furyl (9e) and pyridyl (9f), were a consequence of challenges in purification by column chromatography. The low yields observed for anilines (9m and 9n) were attributed to the lower nucleophilicity of anilines compared to aliphatic amines, and therefore likely incomplete S_N_2 reaction under these conditions, and similarly when reaction was attempted with *tert*-butyl carbamate no desired product (9o) was obtained, with starting material recovered. Reaction with cadaverine yielded a “dimeric” product resulting from cyclisation at each end of the diamine (9l).

We believe the reaction efficiency is inherently dependent on the nucleophilicity of the amines, and the stability of the products to isolation, and further optimisation may yet be possible if a particular structure is of interest.

## Conclusions

A mild, transition-metal free cyclisation to form isoquinolinones has been demonstrated for a range of amines. In our hands this reaction was found not to need, and in fact to perform better in the absence of, iodine, avoiding sustainability concerns associated with disposal of iodine containing wastes.^36^ Whilst exploration of scope was conducted in DMF, DMSO and MeCN were also seen to give reasonable yields when solvents were explored. It is hoped that the mildness of these conditions, and the potential for this reaction to be extended make this a transformation of interest.

## Author contributions

Hannah Chapman: investigation, conceptualization; Callum Dutton; investigation; Helen Sneddon: conceptualization, methodology, project administration, supervision, writing.

## Conflicts of interest

There are no conflicts to declare.

## Supplementary Material

RA-016-D6RA05642C-s001

## Data Availability

The data supporting this article have been included as part of the supplementary information (SI). Supplementary information: spectral data of isolated compounds, and data underpinning Fig. 5. See DOI: https://doi.org/10.1039/d6ra05642c.
