# Supplementary material for: A transition metal-free route to N-functionalised ester substituted isoquinolinones
Source: RSC Adv. 2026 Jul 3;16(35):36464–8. doi: 10.1039/d6ra05642c (PMC13330780; doi:10.1039/d6ra05642c)
Supplement: RA-016-D6RA05642C-s001 [file RA-016-D6RA05642C-s001.pdf]

Supplementary Information (SI) for RSC Advances.
This journal is © The Royal Society of Chemistry 2026

A transition metal-free route to *N*-functionalised ester substituted isoquinolinones

Hannah Chapman, [a] Callum Dutton [a] and Helen F. Sneddon *[a]

Green Chemistry Centre of Excellence, Department of Chemistry,
University of York
Heslington, York, YO10 5DD, UK
E-mail: helen.sneddon@york.ac.uk

Table of Contents

General Information 2

Synthesis of methyl (*E*)-2-(2-(bromomethyl)phenyl)-3-methoxyacrylate (**2**) 3

Serendipitous discovery of an air-mediated isoquinolinone synthesis:

Isolation of methyl 1-oxo-2-(2-(2,2,3-trimethylcyclopent-3-en-1-yl)ethyl)-1,2-dihydroisoquinoline-4-carboxylate (**4**) 5

Identification of dihydroisoquinolinone intermediate **8** 6

Condition Screening Monitored by Gas Chromatography (includes characterisation data for **9a**) 7

General Procedure for Isoquinolinone Synthesis 10

Characterisation data for **9b-9m** 11

Spectra 17

General Information

All reagents and solvents were purchased from commercial suppliers and used without further purification. Solvents were generally not dried, unless mentioned, in which case they were dried using PureSolv MD 7 solvent purification system, Inertcorp. TLC analyses were carried out using silica gel TLC plates and visualised by UV. Column chromatography was carried out on silica gel where the mobile phase was a gradient of 0% ethyl acetate in hexane to 20% ethyl acetate in hexane, increasing in 5% increments unless otherwise stated. All NMR characterisation was carried out by dissolving the sample in $CDCl_3$ and were carried out using a Jeol ECS400 NMR spectrometer. The chemical shifts of NMR samples were quoted in parts per million relative to $CHCl_3$ (δ_H 7.26) and $CDCl_3$ (δ_C 77.0), the central line of the triplet. All IR spectra were recorded using ATR-IR spectroscopy on a Perkin-Elmer FT-IR Spectrum Two spectrometer. All melting points were determined using a Stuart SMP20 instrument. High-resolution mass spectrometry was performed by the University of York Mass Spectrometry Service, using Electrospray Ionisation (ESI). For GC, the Agilent Technologies HP 6890 gas chromatograph was used, with a flame ionisation detector, fitted with an Rxi-5HT capillary column (30m, 250μmx0.25μm nominal, max temperature 260 °C). Helium was used as the carrier gas, at a flow rate of 15 mL/min, a split rate of 15:1 and a 1 μL injection. The oven's starting temperature was 50 °C, which was increased at a rate of 30 °C/min, where it was held for 5 min. The total run time was 11.7 min. Injection temperature and the detector temperature were both 250 °C.

Synthesis of methyl (*E*)-2-(2-(bromomethyl)phenyl)-3-methoxyacrylate (2)

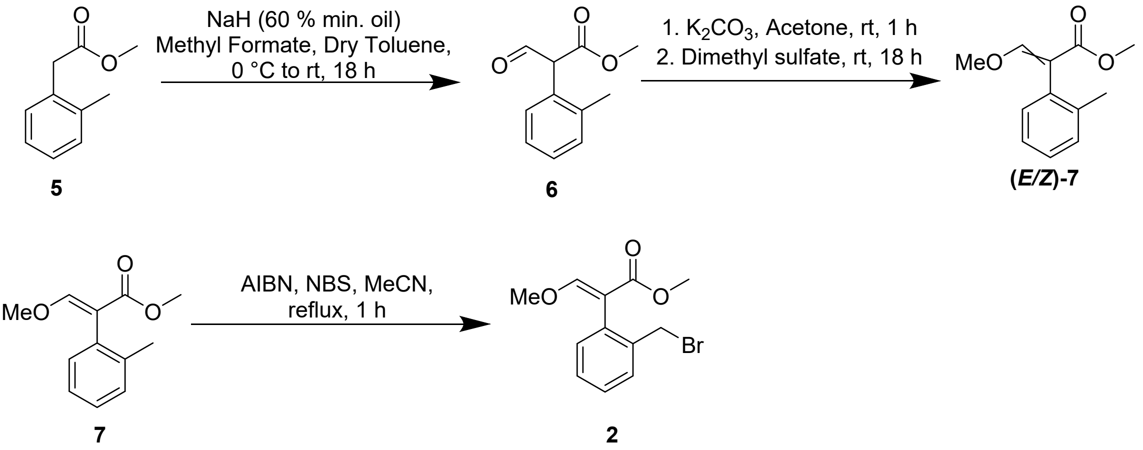

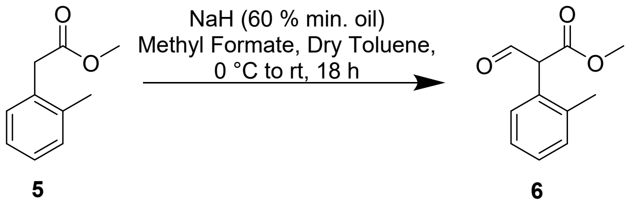

In a 500 mL round-bottom flask under nitrogen, methyl-*o*-tolylacetate (9.26 mL, 10.0 g, 0.06 mol, 1.0 eq.) was added to dry toluene (100 mL), cooled to 0 °C and allowed to stir. Methyl formate (56.2 mL, 55.0 g, 0.915 mol, 15.0 eq.) was then added along with NaH in 60% mineral oil (4.87 g, 0.122 mol, 2.0 eq.) portion wise and allowed to stir overnight to give a white suspension. The product suspension was then extracted into water, which was then acidified with 2M HCl (approx. 10 mL) until pH = 3 is reached. The aqueous layer is then extracted with EtOAc (3 x 30 mL) and the organic layer is then dried over anhydrous $MgSO_4$, filtered and the resultant filtrate was concentrated in vacuo to give a colourless oil (9.08 g, 0.0467 mol, 79%).

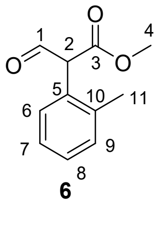

^{1}H NMR (400 MHz; $CDCl_3$) δ_H 11.88 (d, *J* = 12.7 Hz, 1 H, H1), 7.25–7.08 (m, 5 H, H2, H6, H7, H8, H9), 3.74 (s, 3 H, H4), 2.20 (s, 3 H, H11) ppm. **^{13}C NMR** (100 MHz; $CDCl_3$) δ_C 171.37, 163.08, 138.38, 133.19, 131.22, 129.93, 128.11, 125.80, 107.40, 51.80, 21.05 ppm. **IR** (ATR) v_{max}/cm^{-1}: 3014w (aromatic C-H), 2982m (aliphatic C-H), 2954m (aliphatic C-H), 2847m (aliphatic C-H), 1737s (ester C=O), 1702s (aldehyde C=O), 1248s (C-O), 1158s (C-O). **HRMS** (ESI) m/z: [M + Na]$^+$ Calc. 215.0684; found 215.0665.

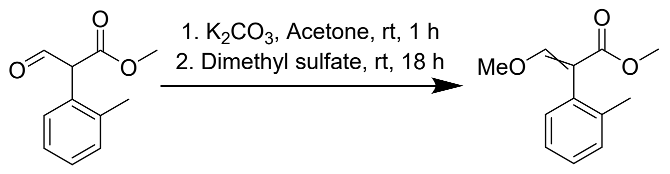

6 **(*E/Z*)-7**

Methyl 3-oxo-2-o-tolylpropanoate (3.81 g, 19.8 mmol, 1.0 equiv.) and K_2CO_3 (4.11 g, 29.8 mmol, 1.5 equiv.) were stirred in acetone (50 mL) for 1 h at r.t. under a nitrogen environment. Dimethyl sulfate (2.82 mL, 29.8 mmol, 1.5 equiv.) was then added and the reaction was stirred at rt. for 24 hrs. Upon completion, the reaction was filtered and the solvent evaporated under reduced pressure. The residue was redissolved in ethyl acetate (approx. 40 mL) and washed with saturated $NaHCO_3$ (approx. 20 mL), water (approx. 20 mL) and then saturated brine (approx. 40 mL). The organic layer was dried over anhydrous $MgSO_4$ and concentrated under vacuum to a yellow oil (4.27 g). Purification over flash chromatography column on silica gel yielded the desired product as a clear oil (3.23 g, 79%).

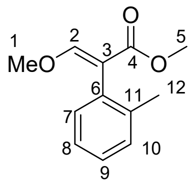

(*E*)-7

^{1}H NMR (400 MHz; $CDCl_3$) δ_H 7.57 (s, 1 H, H2), 7.24–7.16 (m, 3 H, H8, H9 and H10), 7.13–7.09 (m, 1 H, H7), 3.82 (s, 3 H, H5), 3.70 (s, 3 H, H1), 2.19 (s, 3 H, H12) ppm. **^{13}C NMR** (100 MHz; $CDCl_3$) δ_C 168.37 (C4), 159.72 (C2), 137.32, 132.33, 130.72, 129.91, 127.91, 125.52, 111.33 (C3), 61.94 (C1), 51.70 (C5), 19.82 (C12) ppm. **IR** (ATR) ν_{max}/cm^{-1}: 3020 (aromatic C-H), 2949 (aliphatic C-H), 2847 (aliphatic C-H), 1703 (C=O), 1630 (C=C), 1251 (C-O), 1126 (C-O), 1106 (C-O). **HRMS** (ESI) m/z: $[M + Na]^+$ Calc. 229.0841; found 229.0835. Characterisation data matches data found in the literature.[i, ii]

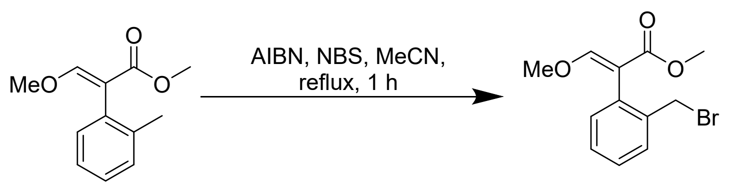

7 **2**

2,2′-Azobis(2-methylpropionitrile) (143 mg, 0.87 mmol, 0.1 equiv.) and *N*-bromosuccinamide (1.87 g, 10.48 mmol, 1.2 equiv.) was added to a solution of (*E*)-methyl-2-(methylphenyl)-3-methoxyacrylate (1.80 g, 8.73 mmol, 1.0 equiv.) in acetonitrile (20 mL) and stirred at reflux for 1 hr. Over time the reaction changed from a yellow to a brown solution. After completion, the solvent was evaporated under reduced pressure; then the residue was extracted into ethyl acetate (approx. 50 mL) and washed twice with water (approx. 40 mL) and then brine (approx. 20 mL). The organic layer was dried over anhydrous $MgSO_4$ and concentrated under vacuum to an oily-orange solid. Purification by flash chromatography

column on silica gel to afford a an off-white solid, which was then triturated with a 4/1, hexane-ethyl acetate mixture the to obtain the desired product as a white powder (4.12 g, 69%).

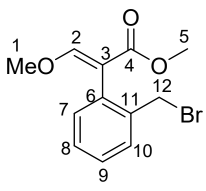

2

mp. 97–99 °C. **R_F value** = 0.53 (ethyl acetate:hexane, 2:3). **^{1}H NMR** (400 MHz; $CDCl_3$) δ_H 7.64 (s, 1 H, H2), 7.49–7.46 (m, 1 H, H10), 7.35–7.31 (m, 2 H, H8 and H9), 7.15–7.11 (m, 1 H, H7), 4.41 (s, 2 H, H12), 3.84 (s, 3 H, H1), 3.71 (s, 3 H, H5) ppm. **^{13}C NMR** (100 MHz; $CHCl_3$) δ_C 167.89 (C4), 160.62 (C2), 136.66, 132.70, 131.53, 130.33, 128.55, 128.53, 109.85 (C3), 62.10 (C1), 51.83 (C5), 32.00 (C12) ppm. **IR** (ATR) v_{max}/cm^{-1}: 3003, (aromatic C-H), 2973 (aliphatic C-H), 2937 (aliphatic C-H), 2846 (aliphatic C-H), 1698 (C=O), 1624 (C=C), 1254 (C-O), 1128 (C-O), 1088 (C-O), 597 (C-Br). **HRMS** (ESI) m/z: [M + Na]$^+$ Calc. 306.9946; found 306.9932. Characterisation data matches data found in the literature.[i, ii, iii, iv, v]

Serendipitous discovery of an air-mediated isoquinolinone synthesis – Isolation of methyl 1-oxo-2-(2-(2,2,3-trimethylcyclopent-3-en-1-yl)ethyl)-1,2-dihydroisoquinoline-4-carboxylate (4)

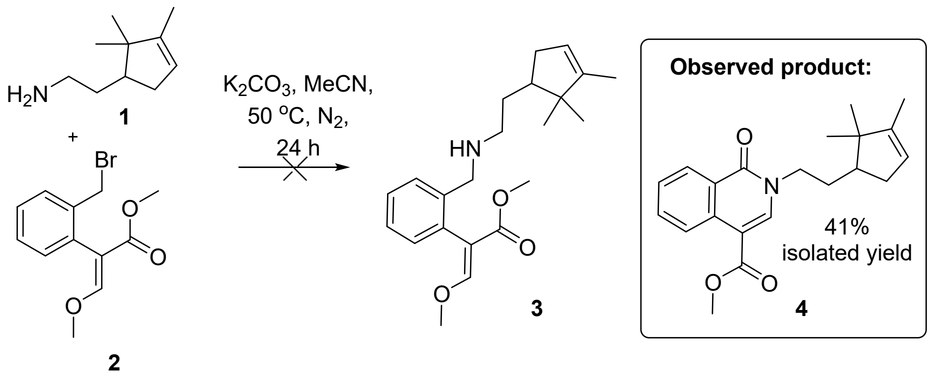

In a 30 mL clear vial, (*E*)-methyl 2-(2-(bromomethyl)-phenyl)-3-methoxyacrylate (150 mg, 0.53 mmol, 1.00 equiv.), *α*-campholenic amine[1] (105 mg, 0.69 mmol, 1.30 equiv.) and anhydrous potassium carbonate (95 mg, 0.69 mmol, 1.30 equiv.) were suspended in acetonitrile (2 mL) under an N_2 environment. The reaction mixture was stirred at 50°C for 18 hrs. After cooling, the solvent was removed under vacuum and re-extracted into EtOAc (approx. 15 mL) and washed with saturated $NaHCO_3$ (approx. 3 x 10 mL) and then brine (approx. 10 mL). The organic layer was dried over anhydrous $MgSO_4$ and concentrated under vacuum to a yellow oil. The crude was purified by two subsequent flash chromatography columns on silica gel to afford the final product as a colourless oil (70 mg, 41%).

[1] Reagent synthesised by Megan Goss.

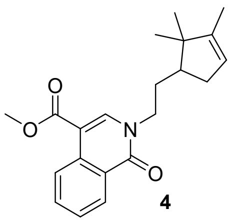

4

$\mathbf{R_F}$ value = 0.56 (ethyl acetate/hexane, 2/3). **^{1}H NMR** (400 MHz $CDCl_3$) δ 8.80 (d, *J* = 8.4 Hz, 1H), 8.45 (d, *J* = 8.4 Hz, 1H), 8.16 (s, 1H), 7.78 – 7.68 (m, 1H), 7.57 – 7.49 (m, 1H), 5.24 (s, 1H), 4.15 – 3.98 (m, 2H), 3.93 (s, 3H), 2.45 – 2.34 (m, 1H), 2.06 – 1.54 (m, 7H), 0.99 (s, 3H), 0.78 (s, 3H) ppm. **^{13}C NMR** (101 MHz $CDCl_3$) δ 165.87, 162.27, 148.63, 140.00, 134.39, 133.12, 128.16, 127.34, 125.56, 125.40, 121.53, 106.72, 51.97, 50.04, 47.91, 47.23, 35.57, 30.20, 25.96, 19.97, 12.73 ppm. **ATR-FTIR:** 3086, 3039, 2952, 2895, 2866, 1714, 1654, 1619, 1556, 1488, 1434, 1403, 1382, 1362, 1310, 1284, 1246, 1191, 1178, 1139, 1102, 1078, 1049, 1030, 913, 783, 731, 696 cm^{-1}. **HRMS (ESI)** m/z $[M + Na]^+$ Calc 362.1727 for $C_{21}H_{25}NNaO_3$; Found 362.1731.

Identification of dihydroisoquinolinone intermediate (8)

Attempts to isolate the dihydroisoquinolinone **8** gave a mass spectrum consistent with the structure, and an NMR showing the dihydoisoquinolinone alongside the isoquinolinone product that forms on oxidation (see **4** for pure spectrum of isoquinoline).

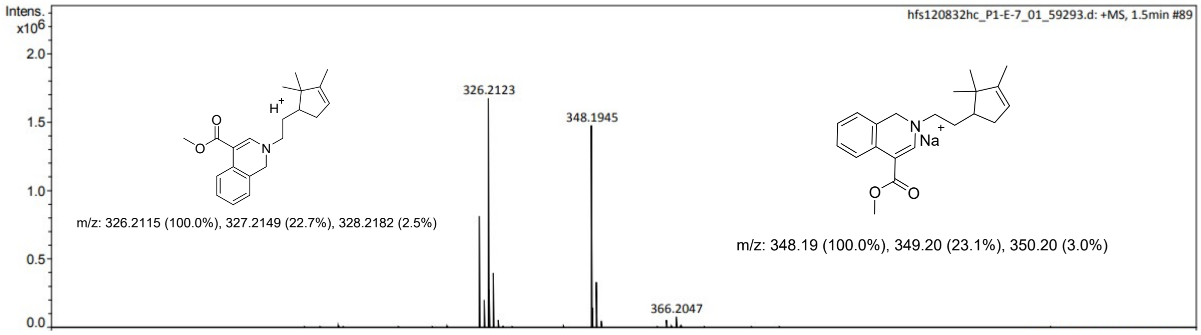

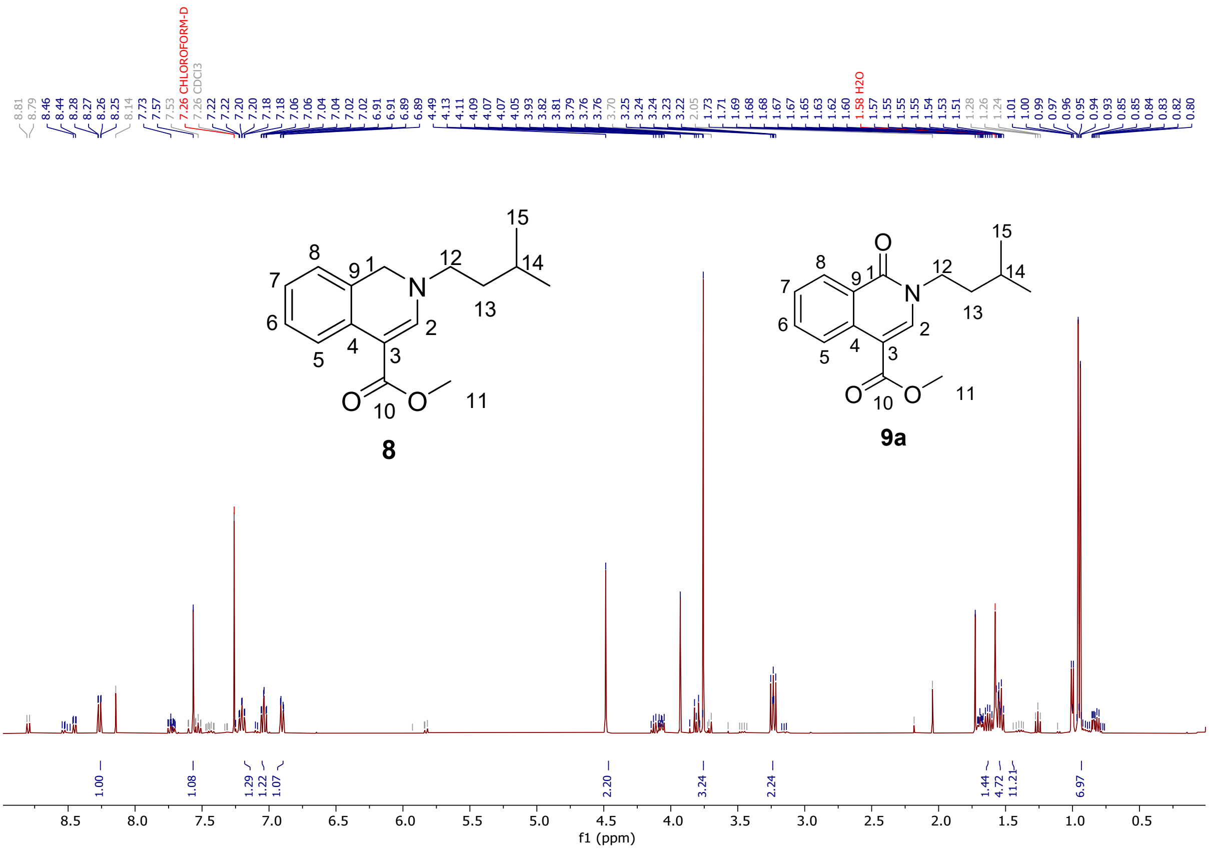

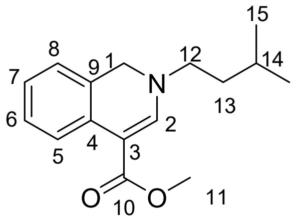

8

^{1}H NMR (400 MHz; $CDCl_3$) δ_H 8.26 (dd, *J* = 8.0, 1.2 Hz, 1 H, H8), 7.56 (s, 1 H, H2), 7.20 (m, 1 H, H7), 7.04 (dd, *J* = 7.4, 7.3 Hz, 1 H, H6), 6.90 (dd, *J* = 7.4, 1.4 Hz, 1 H, H5), 4.49 (s, *2* H, H1), 3.76 (s, 3 H, H11), 3.24 (dd, J = 8.1, 8.0 Hz, 2 H, H12), 1.62 (m, 1 H, H14), 1.54 (m, 2 H, H13), 0.95 (d, *J* = 6.4 Hz, 6 H, H15) ppm

Condition Screening Monitored by Gas Chromatography

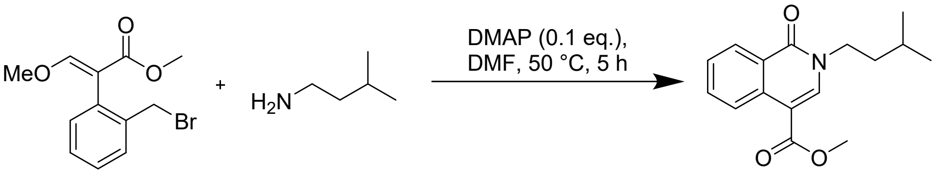

2 **9a**

A 25 mL round-bottom flask was charged with a stirrer bar, methyl (*E*)-2-(2-bromomethylphenyl)-3-methoxyacrylate (100 mg, 0.35 mmol, 1.0 eq.), isoamylamine (0.053 mL, 40 mg, 0.46 mmol, 1.3 eq.), 4-dimethylaminopyridine (56 mg, 0.46 mmol, 1.3 eq.) and dimethyl formamide (2 mL). This was allowed to stir at 50 °C for 18 h before being concentrated under reduced pressure. This gave a brown oily solid as the crude product (163.5 mg). This was extracted into ethyl acetate (10 mL), washed with water (10 mL) and saturated brine solution (10 mL), dried over anhydrous $MgSO_4$, filtered and concentrated before being purified by flash column chromatography on silica gel to give a white solid (23.7 mg, 0.0868 mmol, 25%).

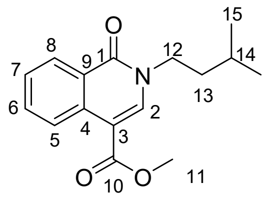

9a

mp. 95–97 °C. **R_F value** = 0.64 (ethyl acetate:hexane 2:3). **^{1}H NMR** (400 MHz; $CDCl_3$) δ_H 8.78 (dd, *J* = 8.4, 1.0 Hz, 1 H, H8), 8.44 (dd, *J* = 8.0, 1.6 Hz, 1 H, H5), 8.13 (s, 1 H, H2), 7.72 (ddd, *J* = 8.4 Hz, 7.1, 1.6 Hz, 1 H, H7), 7.52 (ddd, *J* = 8.0, 7.1, 1.0 Hz, 1 H, H6), 4.05 (t, *J* = 7.6 Hz, 2 H, H12), 3.92 (s, 3 H, H11), 1.71–1.65 (m, 3 H, H13 and H14), 0.99 (d, *J* = 6.5 Hz, 6 H, H15) ppm. **^{13}C NMR** (100 MHz; $CHCl_3$) δ_C 165.86 (C10), 162.27 (C1), 139.98 (C2), 134.36 (C4), 133.11 (C7), 128.15 (C5), 127.32 (C6), 125.57 (C9), 125.38 (C8), 106.69 (C3), 51.95 (C11), 48.64 (C12), 38.37 (C13), 26.09 (C14), 22.57 (C15) ppm. **IR** (ATR) v_{max}/cm^{-1}: 3059w (aromatic C-H), 2999m (aliphatic C-H), 2954m (aliphatic C-H), 2872m (aliphatic C-H), 1713s (ester C=O), 1658vs (lactam C=O), 1619s (C=C), 1181s (C-O), 1030s (C-O). **HRMS** (ESI) m/z: [M + Na]$^+$ Calc. 296.1263; found 296.1257.

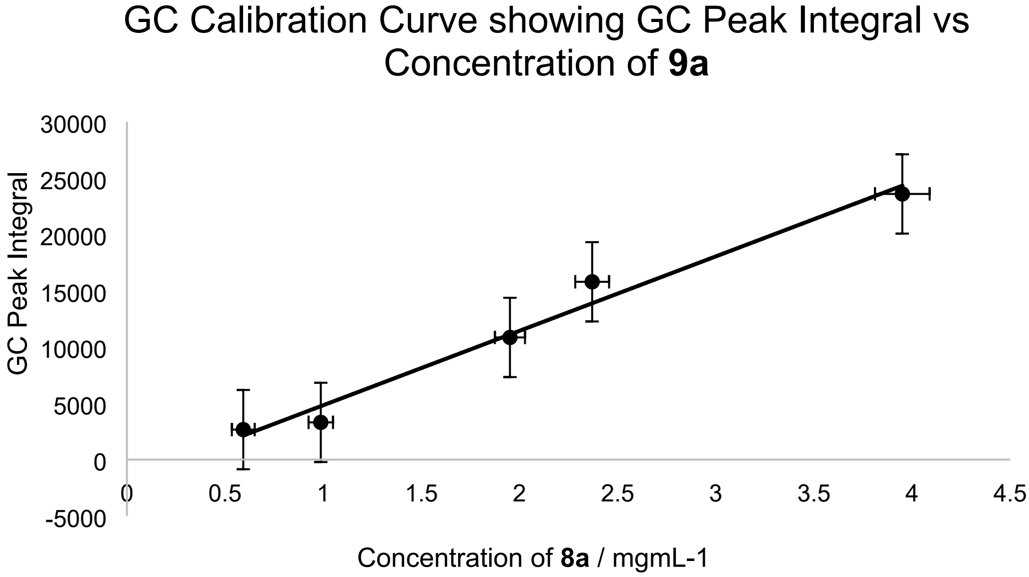

A calibration curve was then formed from screening serial dilutions of the isoquinolinone product, where the 23.7 mg product was dissolved in 4 mL acetonitrile, and 1 mL, 0.6 mL, 0.5 mL, 0.25 mL and 0.15 mL were separately extracted and each made up to 1.5 mL in a GC vial. Following this, the mass of isoquinolinone in each 1.5 mL vial was calculated, and a GC experiment on each solution was carried out. The peak area was then plotted against the mass of the isoquinolinone product to give a calibration curve. Errors for the concentration of each solution were calculated from the errors of the syringes used to form each of the standard solutions which were propagated in quadrature to give a total percentage error of the volume of solvent and the error in the mass was found from the mass balance, found as a percentage and then propagated through quadrature when considering dilutions as the error associated with the mass grows with each dilution and then percentage errors in concentration were found from both the percentage error in volume of solvent and the mass of **9a**, which were then multiplied by the theoretical concentrations of each of the standard solutions to give their absolute errors which are plotted parallel to the x-axis. The standard error of each measurement is plotted on the y-axis to show the limitations of using this linear regression to model the GC peak integrals and concentration of **9a**.

$$Total\ Percentage\ Error = \sqrt{\left(\frac{\delta a}{a}\right)^2 + \left(\frac{\delta b}{b}\right)^2 + \ldots}$$

Where δa is the absolute uncertainty of a quantity and a is the value of that quantity is used to propagate percentage errors in quadrature.

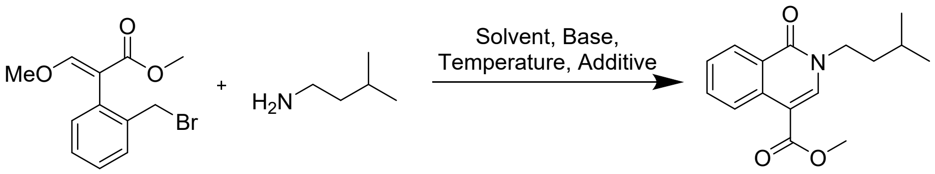

A test tube was charged with a stirrer bar, methyl (*E*)-2-(2-bromomethylphenyl)-3-methoxyacrylate (50 mg, 0.175 mmol, 1.0 eq.), isoamylamine (0.027 mL, 20 mg, 0.263 mmol, 1.5 eq), the base and the additive. These were then dissolved in solvent (2 mL), heated to the required temperature and allowed to stir over 5 hours under reflux. During the reaction, 0.15 mL aliquots of the reaction mixtures were extracted hourly, filtered through a PTFE membrane into a GC vial, which is then made up to 1.5 mL using acetonitrile and monitored by gas chromatography. Approximate conversions were then calculated using the calibration curve and integrals of the peak associated with the isoquinolinone product, where the equation of the calibration curve was rearranged to give masses in each GC vial.

$$Mass\ in\ 1.5\ mL\ GC\ vial = \frac{Peak\ Area + 1784.8}{4408.3}$$

These masses were then added together for the first four aliquots and then for the final aliquot, it was assumed 1.4 mL of solvent remained (as the reaction vessel was under reflux) in the test tube so the final concentration was multiplied by 1.4 and added to the first four aliquots to give an overall conversion.

Solvent	dD	dP	dH	conversion
MeCN	15.3	18	6.1	33
DMF	17.4	13.7	11.3	100
EtOAc	15.8	5.3	7.2	45
DMSO	18.4	16.4	10.2	80
Toluene	18	1.4	2	29
DMC	15.5	8.6	9.7	14
THF	16.8	5.7	8	42
GVL	16.8	16.5	6.7	25
2-MeTHF	16.9	5	4.3	36
Chlorobenzene	19	4.3	2	49
DCE	18	7.4	4.1	49
Cyclopentanone	17.9	11.9	5.2	14
CHCl3	17.8	3.1	5.7	37
NMP	18	12.3	7.2	81
MeOH	14.7	12.3	22.3	47
EtOH	15.8	8.8	19.4	31
nBuOH	16	5.7	15.8	36

General Procedure for Isoquinolinone Synthesis

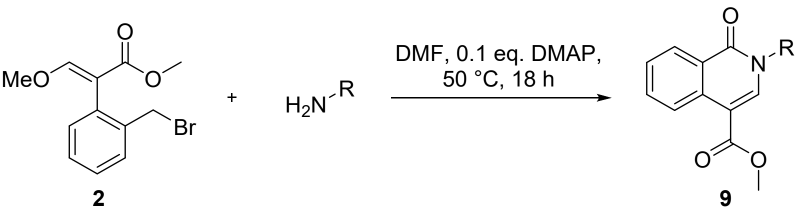

A 25 mL round-bottom flask is charged with a stirrer bar followed by methyl (*E*)-2-(2-bromomethylphenyl)-3-methoxyacrylate (100 mg, 0.35 mmol, 1.0 eq.), amine (0.46 mmol, 1.3 eq.) and 4-dimethylaminopyridine (4.3 mg, 0.035 mmol, 0.10 eq.) followed by dimethyl formamide (4 mL). This was then heated to 50 °C and allowed to stir for 18 h. The reaction mixture was then extracted into ethyl acetate (10 mL) and washed with water (2 x 10 mL) and saturated brine solution (10 mL), dried over anhydrous $MgSO_4$, filtered and concentrated under a vacuum to give a crude product. This was then purified by flash column chromatography on silica gel to give the product.

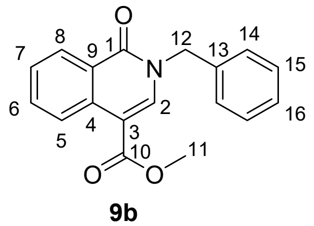

9b

Gave a white powder as the product (36.8 mg, 0.126 mmol, 36%). **mp.** 138–140 °C. **R_F value** = 0.62 (ethyl acetate:hexane, 1:1). **^{1}H NMR** (400 MHz; $CDCl_3$) δ_H 8.81 (dd, *J* = 9.2, 1.0 Hz, 1 H, H8), 8.49 (dd, *J* = 8.1, 1.5 Hz, 1 H, H5), 8.19 (s, 1 H, H2), 7.75 (ddd, *J* = 8.5, 7.2, 1.2 Hz, 1 H, H7), 7.55 (ddd, *J* = 8.2, 7.2, 1.3 Hz. 1 H, H6), 7.38–7.28 (m, 5 H, H14, H15 and H16), 5.27 (s, 2 H, H12), 3.89 (s, 3 H, H11). **^{13}C NMR** (100 MHz; $CDCl_3$) δ_C 165.71 (C10), 162.43 (C1), 139.74 (C2), 136.19 (C13), 134.39 (C4), 133.34 (C7), 129.13 (C14), 128.43 (C5), 128.34 (C16), 128.10 (C15), 127.51 (C6), 125.62 (C9), 125.52 (C8), 107.19 (C3), 52.65 (C12), 51.99 (C11) ppm. **IR** (ATR) v_{max}/cm^{-1}: 3079w (aromatic C-H), 3052w (aromatic C-H), 2944m (aliphatic C-H), 2843m (aliphatic C-H), 1715s (ester C=O), 1645s (lactam C=O), 1616s (C=C), 1176s (C-O), 1028s (C-O). **HRMS** (ESI) m/z: [M + Na]$^+$ Calc. 316.0950; found 316.0954. Characterisation data matches data found in the literature.[vi]

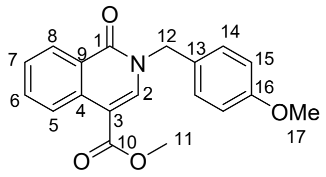

9c

Gave a white powder as the product (55.5 mg, 0.172 mmol, 49%). **mp.** 141–143 °C. **R_F value** = 0.53 (ethyl acetate:hexane, 1:1). **^{1}H NMR** (400 MHz; $CDCl_3$) δ_H 8.79 (dd, *J* = 8.4, 0.6 Hz, 1 H, H8), 8.48 (dd, *J* = 8.2, 1.5 Hz, 1 H, H5), 8.18 (s, 1 H, H2), 7.73 (ddd, *J* = 8.2, 7.1, 1.5 Hz, 1 H, H7), 7.54 (ddd, *J* = 8.2, 7.1, 1.2 Hz, 1 H, H6), 7.31 (d, *J* = 8.8 Hz, 2 H, H14), 6.88 (d, *J* = 8.8 Hz, 2 H, H15), 5.19 (s, 2 H, H12), 3.89 (s, 3 H, H11), 3.79 (s, 3 H, H17). **^{13}C NMR** (100 MHz; $CDCl_3$) δ 165.74 (C10), 162.42 (C1), 159.65 (C16), 139.62 (C2), 134.35 (C4), 133.27 (C7), 129.72 (C14), 128.37 (C5), 128.28 (C13), 127.44 (C6), 125.62 (C9), 125.47 (C8), 114.46 (C15), 107.06 (C3), 55.44 (C17), 52.17 (C12), 51.98 (C11). **IR** (ATR) v_{max}/cm^{-1}: 3051w (aromatic C-H), 3003w (aromatic C-H), 2950m (aliphatic C-H), 2842m (aliphatic C-H), 1705s (ester C=O), 1661s (lactam C=O), 1607s (C=C), 1246s (C-O), 1179s (C-O), 1099s (C-O), 1024s (C-O). **HRMS** (ESI) m/z: [M + Na]$^+$ Calc. 346.1055; found 346.1056.

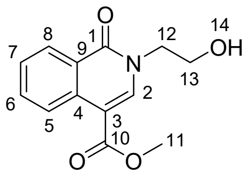

9d

Gave white needles as the product (43.5 mg, 0.176 mmol, 50%). **mp.** 140–142 °C. **R_F value** = 0.16 (ethyl acetate:hexane, 1:1). **^{1}H NMR** (400 MHz; $CDCl_3$) δ_H 8.84 (dd, J = 8.5, 0.9 Hz, 1 H, H8), 8.45 (dd, J = 8.2, 1.8 Hz, 1 H, H5), 8.22 (s, 1 H, H2), 7.75 (ddd, J = 8.5, 7.1, 1.8 Hz, 1 H, H7), 7.55 (ddd, J = 8.2, 7.1, 0.9 Hz, 1 H, H6), 4.25 (t, J = 5.3 Hz, 2 H, H12), 4.03 (t, J = 5.3 Hz, 2 H, H13), 3.92 (s, 3 H, H11). OH not observed.**^{13}C NMR** (100 MHz; $CDCl_3$) δ 165.71 (C10), 162.54 (C1), 141.42 (C2), 134.48 (C4),

132.99 (C7), 127.77 (C5), 126.98 (C6), 125.17 (C8), 125.13 (C9), 105.94 (C3), 60.30 (C13), 52.86 (C12), 51.75 (C11). **IR** (ATR) v_{max}/cm^{-1}: 3386br (O-H), 3062w (aromatic C-H) 2972m (aliphatic C-H), 2947m (aliphatic C-H), 2856m (aliphatic C-H), 1713s (ester C=O), 1633s (lactam (C=O), 1596s (C=C), 1179s (C-O), 1075s (C-O), 1030s (C-O). **HRMS** (ESI) m/z: $[M + Na]^+$ Calc. 270.0742; found 270.0735.

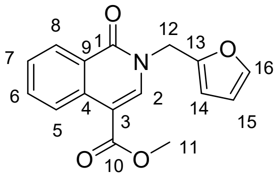

9e

Gave a white powder as the product (34.3 mg, 0.121 mmol, 35%). **mp.** 137–138 °C. **R_F value** = 0.58 (ethyl acetate:hexane, 1:1). **^{1}H NMR** (400 MHz; $CDCl_3$) δ_H 8.80 (ddd, *J* = 8.5, 1.2, 0.7 Hz, 1 H, H8), 8.46 (ddd, *J* = 8.1, 1.5, 0.7 Hz, 1 H, H5), 8.26 (s, 1 H, H2), 7.74 (ddd, *J* = 8.5, 7.1, 1.5 Hz, 1 H, H7), 7.53 (ddd, *J* = 8.1, 7.1, 1.2 Hz, 1 H, H6), 7.40 (dd, *J* = 1.9, 0.8 Hz, 1 H, H16), 6.47 (dd, *J* = 3.3, 0.7 Hz, 1 H, H14), 6.36 (dd, *J* = 3.3, 1.9 Hz, 1 H, H15), 5.23 (s, 2 H, H12), 3.92 (s, 3 H, H11). **^{13}C NMR** (100 MHz; $CDCl_3$) δ_C 165.71 (C10), 162.05 (C1), 148.93 (C13), 143.28 (C16), 139.40 (C2), 134.37 (C4), 133.35 (C7), 128.32 (C5), 127.46 (C6), 125.49 (C8 and C9 believed to overlap), 110.89 (C15), 110.13 (C14), 107.11 (C3), 52.01 (C11), 45.18 (C12) ppm. **IR** (ATR) v_{max}/cm^{-1}: 3119w (aromatic C-H), 3081w (aromatic C-H), 2999w (aromatic C-H), 2946m (aliphatic C-H), 2843m (aliphatic C-H), 1721s (ester C=O), 1644s (lactam C=O), 1618s (C=C), 1603s (furan C=C), 1487s (furan (C=C), 1178s (C-O), 1143s (furan C-O), 1050s (furan C-O), 1027s (C-O). **HRMS** (ESI) m/z: $[M + Na]^+$ Calc. 306.0742; found 306.0748.

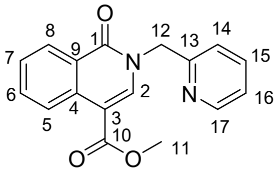

9f

Gave a white powder (30.2 mg, 0.103 mmol, 29%). **mp.** 130–132 °C. **R_F value** = 0.57 (dichloromethane:methanol:triethylamine, 95:4:1). **^{1}H NMR** (400 MHz; $CDCl_3$) δ_H 8.83 (ddd, *J* = 8.5, 1.1, 0.6 Hz, 1 H, H8), 8.56 (ddd, *J* = 4.8, 1.8, 1.1 Hz, 1 H, H17), 8.44 (ddd, *J* = 8.1, 1.4, 0.6 Hz, 1 H, H5), 8.40 (s, 1 H, H2), 7.74 (ddd, *J* = 8.5, 7.2, 1.4 Hz, 1 H, H7), 7.67 (ddd, *J* = 7.7, 7.7, 1.8 Hz, 1 H, H15), 7.52 (ddd, *J* = 8.1, 7.2, 1.1 Hz, 1 H, H6), 7.40 (m, 1 H, H14), 7.22 (ddd, *J* = 7.7, 4.8, 1.1 Hz, 1 H, H16), 5.33 (s, 2 H, H12), 3.90 (s, 3 H, H11) ppm. **^{13}C NMR** (100 MHz; $CDCl_3$) δ_C 165.72 (C10), 162.34 (C1), 155.44 (C13), 149.78 (C17), 140.56 (C2), 137.18 (C15), 134.61 (C4), 133.32 (C7), 128.23 (C5), 127.37 (C6), 125.50 (C8), 125.45 (C9), 123.15 (C16), 123.03 (C14), 106.99 (C3), 54.63 (C12), 51.92 (C11) ppm. **IR** (ATR) v_{max}/cm^{-1}: 3015w (aromatic C-H), 2955m (aliphatic C-H), 2918m (aliphatic C-H), 2849m (aliphatic C-H), 1713s (ester C=O), 1651s (lactam C=O), 1617s (C=C), 1590s (C=N), 1568m (C=C), 1150s (C-O), 1028s (C-O). **HRMS** (ESI) m/z: $[M + Na]^+$ Calc. 317.0902; found 317.0897.

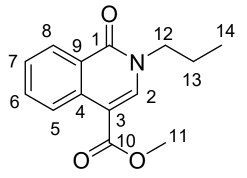

9g

Gave a white powder as the product (45.1 mg, 0.184 mmol, 52%). **mp.** 64.0–65.0 °C. **R_F value** = 0.52 (ethyl acetate:hexane, 1:1). **^{1}H NMR** (400 MHz; $CDCl_3$) δ_H 8.80 (ddd, *J* = 8.5, 1.2, 0.7 Hz, 1 H, H8), 8.46 (ddd, *J* = 8.1, 1.5, 0.7 Hz, 1 H, H5), 8.15 (s, 1 H, H2), 7.74 (ddd, *J* = 8.5, 7.1, 1.5 Hz, 1 H, H7), 7.53 (ddd, *J* = 8.1, 7.1, 1.2 Hz, 1 H, H6), 4.02 (t, *J* = 7.3 Hz, 2 H, H12), 3.92 (s, 3 H, H11), 1.85 (m, 2 H, H13), 1.00 (t, *J* = 7.4 Hz, 3 H, H14) ppm. **^{13}C NMR** (100 MHz; $CDCl_3$) δ_C 165.88 (C10), 162.34 (C1), 140.16 (C2), 134.40 (C4), 133.14 (C7), 128.21 (C5), 127.32 (C6), 125.60 (C9), 125.39 (C8), 106.52 (C3), 51.96 (C11), 51.78 (C12), 22.77 (C13), 11.29 (C14) ppm. **IR** (ATR) ν_{max}/cm^{-1}: 3085w (aromatic C-H), 2981m (aliphatic C-H), 2951m (aliphatic C-H), 2851m (aliphatic C-H), 1711s (ester C=O), 1655vs (lactam C=O), 1618s (C=C), 1184s (C-O), 1029s (C-O). **HRMS** (ESI) m/z: $[M + Na]^+$ Calc. 268.0950; found 268.0943.

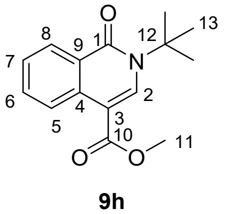

9h

Gave a white powder as the product (33.8 mg, 0.130 mmol, 37%). **mp.** 129–130 °C. **R_F value** = 0.61 (ethyl acetate:hexane, 1:1). **^{1}H NMR** (400 MHz; $CDCl_3$) δ_H 8.73 (ddd, *J* = 8.4, 1.1, 0.7 Hz, 1 H, H8), 8.49 (s, 1 H, H2), 8.43 (ddd, *J* = 8.1, 1.5, 0.7 Hz, 1 H, H5), 7.70 (ddd, *J* = 8.4, 7.1, 1.5 Hz, 1 H, H7), 7.50 (ddd, *J* = 8.2, 7.1, 1.1 Hz, 1 H, H6), 3.92 (s, 3 H, H11), 1.76 (s, 9 H, H13) ppm. **^{13}C NMR** (100 MHz; $CDCl_3$) δ_C 166.39 (C10), 163.26 (C1), 137.30 (C2), 133.82 (C4), 132.90 (C7), 128.16 (C5), 127.00 (C6), 126.97 (C9), 124.86 (C8), 105.73 (C3), 62.10 (C12), 51.89 (C11), 28.82 (C13) ppm. **IR** (ATR) ν_{max}/cm^{-1}: 3016w (aromatic C-H), 2979s (aliphatic C-H), 2956s (aliphatic C-H), 2935s (aliphatic C-H), 1705s (ester C=O), 1656vs (lactam C=O), 1615s (C=C), 1191s (C-O), 1028s (C-O). **HRMS** (ESI) m/z: $[M + Na]^+$ Calc. 282.1106; found 282.1102.

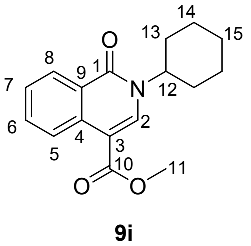

9i

Gave a white powder (43.0 mg, 0.151 mmol, 43%). **mp.** 128–130 °C. **R_F value** = 0.60 (ethyl acetate:hexane, 1:1) **^{1}H NMR** (400 MHz; $CDCl_3$) δ_H 8.78 (ddd, *J* = 8.4 Hz, 1.1, 0.6 Hz, 1 H, H8), 8.46 (ddd, *J* = 8.2, 1.5, 0.6 Hz, 1 H, H5), 8.21 (s, 1 H, H2), 7.71 (ddd, *J* = 8.4, 7.1, 1.5, 1 H, H7), 7.51 (ddd,

J = 8.2, 7.1, 1.1, 1 H, H6), 5.01–4.91 (m, 1 H, H12), 3.92 (s, 3 H, H11), 1.99–1.88 (m, 4 H, H13), 1.81–1.74 (m, 1 H, $H15_{eq}$), 1.64–1.47 (m, 4 H, H14), 1.32–1.20 (m, 1 H, $H15_{ax}$) ppm. **^{13}C NMR** (100 MHz; $CDCl_3$) δ_C 166.02 (C10), 162.07 (C1), 136.26 (C2), 133.90 (C4), 133.11 (C7), 128.44 (C5), 127.22 (C6), 125.49 (C9), 125.24 (C8), 106.64 (C3), 54.61 (C11), 51.96 (C12), 32.59 (C13), 25.94 (C14), 25.47 (C15) ppm. **IR** (ATR) v_{max}/cm^{-1}: 3112w (aromatic C-H), 2981m (aliphatic C-H), 2958m (aliphatic (C-H), 2933s (aliphatic C-H), 2853m (aliphatic C-H), 1711s (ester C=O), 1655vs (lactam C=O), 1617s (C=C), 1188s (C-O), 1028s (C-O). **HRMS** (ESI) m/z: [M + Na]$^+$ Calc. 308.1262; found 308.1257.

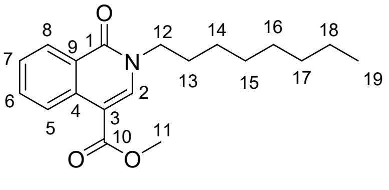

9j

Gave a white oil which solidified when cooled. (36.0 mg, 0.114 mmol, 33%). **mp.** 41.0–43.0 °C. **R_F value** = 0.53 (ethyl acetate:hexane, 1:1). **^{1}H NMR** (400 MHz; $CDCl_3$) δ_H 8.79 (dd, *J* = 8.4, 1.0 Hz, 1 H, H8), 8.45 (dd, *J* = 8.0, 1.5 Hz, 1 H, H5), 8.14 (s, 1 H, H2), 7.72 (ddd, *J* = 8.4, 7.2, 1.5, 1 H, H7), 7.52 (ddd, *J* = 8.0, 7.2, 1.0 Hz, 1 H, H6), 4.04 (t, *J* = 7.5 Hz, 2 H, H12), 3.92 (s, 3 H H11), 1.84–1.75 (m, 2 H, H13), 1.41–1.20 (m, 10 H, H14, H15, H16, H17 and H18), 0.87 (t, *J* = 6.5 Hz, 3 H, H19) ppm. **^{13}C NMR** (100 MHz; $CDCl_3$) δ_C 165.86 (C10), 162.28 (C1), 140.11 (C2), 134.38 (C4), 133.09 (C7), 128.18 (C5), 127.28 (C6), 125.58 (C9), 125.37 (C8), 106.53 (C3), 51.92 (C11), 50.28 (C12), 31.88 (C13), 29.50 (one of C14-C18), 29.32 (one of C14-C18), 29.26 (one of C14-C18), 26.81 (one of C14-C18), 22.73 (one of C14-C18), 14.19 (C19) ppm. **IR** (ATR) v_{max}/cm^{-1}: 3112w (aromatic C-H), 2981m (aliphatic C-H), 2933m (aliphatic C-H), 2853m (aliphatic C-H), 1710s (ester C=O), 1655s (lactam (C=O), 1617s (C=C), 1152s (C-O), 1029s (C-O). **HRMS** (ESI) m/z: [M + Na]$^+$ Calc. 338.1732; found 338.1720.

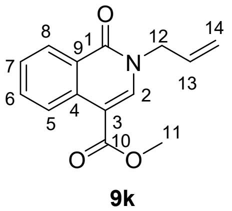

9k

Gave a white powder (57.0 mg, 0.234 mmol, 67%). **mp.** 81.0–83.0 °C. **R_F value** = 0.51 (ethyl acetate:hexane, 1:1). **^{1}H NMR** (400 MHz; $CDCl_3$) δ_H 8.80 (ddd, *J* = 8.5, 1.1, 0.6 Hz, 1 H, H8), 8.47 (ddd, 8.1, 1.5, 0.6 Hz, 1 H, H5), 8.12 (s, 1 H, H2), 7.74 (ddd, *J* = 8.5, 7.1, 1.5 Hz, 1 H, H7), 7.53 (ddd, *J* = 8.1, 7.1, 1.1 Hz, 1 H, H6), 5.99 (ddt, *J* = 17.1, 10.3, 5.8 Hz, 1 H, H13), 5.31 (ddd, *J* = 10.3, 2.4, 1.5 Hz, 1 H, H_E14), 5.26 (ddd, *J* = 17.1, 2.4, 1.5 Hz, 1 H, H_Z14), 4.68 (ddd, *J* = 5.8, 1.5, 1.5 Hz, 2 H, H12), 3.91 (s, 3 H, H11) ppm. **^{13}C NMR** (100 MHz; $CDCl_3$) δ_C 165.72 (C10), 162.11 (C1), 139.51 (C2), 134.37 (C4), 133.26 (C7), 132.18 (C13), 128.28 (C5). 127.42 (C6), 125.49 (C9), 125.44 (C8), 119.08 (C14), 106.96 (C3), 51.96 (C11), 51.47 (C12) ppm. **IR** (ATR) v_{max}/cm^{-1}: 3084w (aromatic C-H), 2981m (aliphatic C-H), 2951m (aliphatic C-H), 2851m (aliphatic C-H), 1710s (ester C=O), 1655s (lactam C=O), 1618s (C=C),

1603s (C=C), 1183s (C-O), 1030s (C-O). **HRMS** (ESI) m/z: [M + Na]$^+$ Calc. 266.0793; found 266.0788.

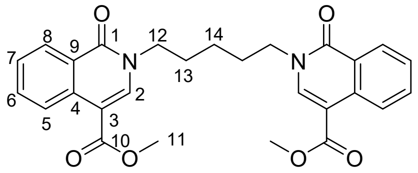

9l

Gave a brown oil (19.0 mg, 0.0402 mmol, 23%). R_F value = 0.20 (ethyl acetate:hexane, 1:1). ^{1}H NMR (400 MHz; $CDCl_3$) δ_H 8.80 (dd, *J* = 8.4, 1.0 Hz, 2 H, H8), 8.43 (dd, *J* = 8.1, 1.3 Hz, 2 H, H5), 8.13 (s, 2 H, H2), 7.74 (ddd, *J* = 8.4, 7.1, 1.3 Hz, 2 H, H7), 7.53 (ddd, *J* = 8.1, 7.1, 1.0 Hz, 2 H, H6), 4.06 (t, *J* = 7.2 Hz, 4 H, H12), 3.92 (s, 6 H, H11), 1.93–1.84 (m, 4 H, H13), 1.53–1.43 (m, 2 H, H14). ^{13}C NMR (100 MHz; $CDCl_3$) δ_C 165.73 (C10), 162.31 (C1), 139.88 (C2), 134.38 (C4), 133.20 (C7), 128.15 (C5), 127.39 (C6), 125.51 (C9), 125.43 (C8), 106.80 (C3), 51.97 (C11), 49.85 (C12), 29.07 (C13), 23.83 (C14) ppm. IR (ATR) v_{max}/cm^{-1}: 3059 (aromatic C-H), 2961 (aliphatic C-H), 2862 (aliphatic C-H), 1713 (ester C=O), 1655 (lactam C=O), 1619 (C=C), 1257 (C-O), 1093 (C-O). HRMS (ESI) m/z: [M -MeOH]$^+$ Calc. 459.16; found 459.1910.

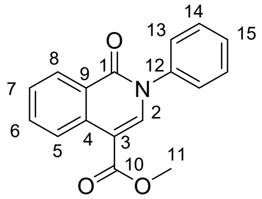

9m

Gave a white powder as the product **(**19.8 mg, 0.0685 mmol, 20%). **mp.** 160–162 °C. **R_F value** = 0.55 (ethyl acetate:hexane, 1:1). **^{1}H NMR** (400 MHz; $CDCl_3$) δ_H 8.87 (m, 1 H, H8), 8.50 (dd, *J* = 8.0, 1.6 Hz, 1 H, H5), 8.26 (s, 1 H, H2), 7.80 (ddd, *J* = 8.5, 7.2, 1.6 Hz, 1 H, H7), 7.60–7.44 (m, 6 H, H6, H13, H14 and H15), 3.90 (s, 3 H, H11) ppm. **^{13}C NMR** (100 MHz; $CDCl_3$) δ_C 165.75 (C10), 162.14 (C1), 140.71 (C12), 140.35 (C2), 134.44 (C4), 133.61 (C7), 129.65 (C13), 128.90 (C15), 128.64 (C5), 127.74 (C6), 126.94 (C14), 125.88 (C9), 125.58 (C8), 107.06 (C3), 52.01 (C11) ppm. **IR** (ATR) v_{max}/cm^{-1}: 3090w (aromatic C-H), 2981m (aliphatic C-H), 2959m (aliphatic C-H), 1713s (ester C=O), 1661s (lactam C=O), 1622s (C=C), 1183s (C-O), 1029s (C-O). **HRMS** (ESI) m/z: [M + Na]$^+$ Calc. 302.0793; found 302.0788. Characterisation data matches data found in the literature.[iii]

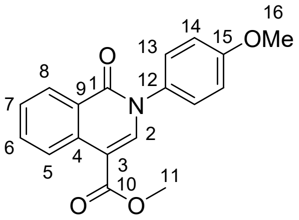

9n

Gave a brown oil (16.4 mg, 0.0530 mmol, 15%). mp. 156–158 °C. R_F value = 0.45 (ethyl acetate:hexane, 1:1). ^{1}H NMR (400 MHz; $CDCl_3$) δ_H 8.86 (dd, *J* = 8.5, 0.9 Hz, 1 H, H8), 8.50 (dd, *J* = 8.0, 1.3 Hz, 1 H, H5), 8.23 (s, 1 H, H2), 7.78 (ddd, *J* = 8.5, 7.2, 1.3 Hz, 1 H, H7), 7.56 (ddd, *J* = 8.0, 7.2, 0.9 Hz, 1 H, H6), 7.35 (d, *J* = 8.9 Hz, 2 H, H14), 7.03 (d, *J* = 8.9 Hz, 2 H, H13), 3.90 (s, 3 H, H11), 3.87 (s, 3 H, H16) ppm. ^{13}C NMR (100 MHz; $CDCl_3$) δ_C 165.74 (C10), 162.35 (C1), 159.68 (C15), 140.59 (C2), 134.40 (C4), 133.46 (C7), 128.56 (C5), 127.96 (C6), 125.78 (C8), 125.49 (C9 or C12), 125.46 (C9 or C12), 114.82 (C13 or C14), 114.70 (C13 or C14), 106.78 (C3), 55.71 (C16), 51.92 (C11) ppm. IR (ATR) v_{max}/cm^{-1}: 3084w (aromatic C-H), 2981m (aliphatic C-H), 2950m (aliphatic C-H), 2852m (aliphatic C-H), 1710s (ester C=O), 1655s (lactam C=O), 1618s (C=C), 1246s (C-O), 1184s (C-O), 1099s (C-O), 1030s (C-O) HRMS (ESI) m/z: $[M + Na]^+$ Calc. 332.0899; found 332.0902. Characterisation data matches data found in the literature. [iii]

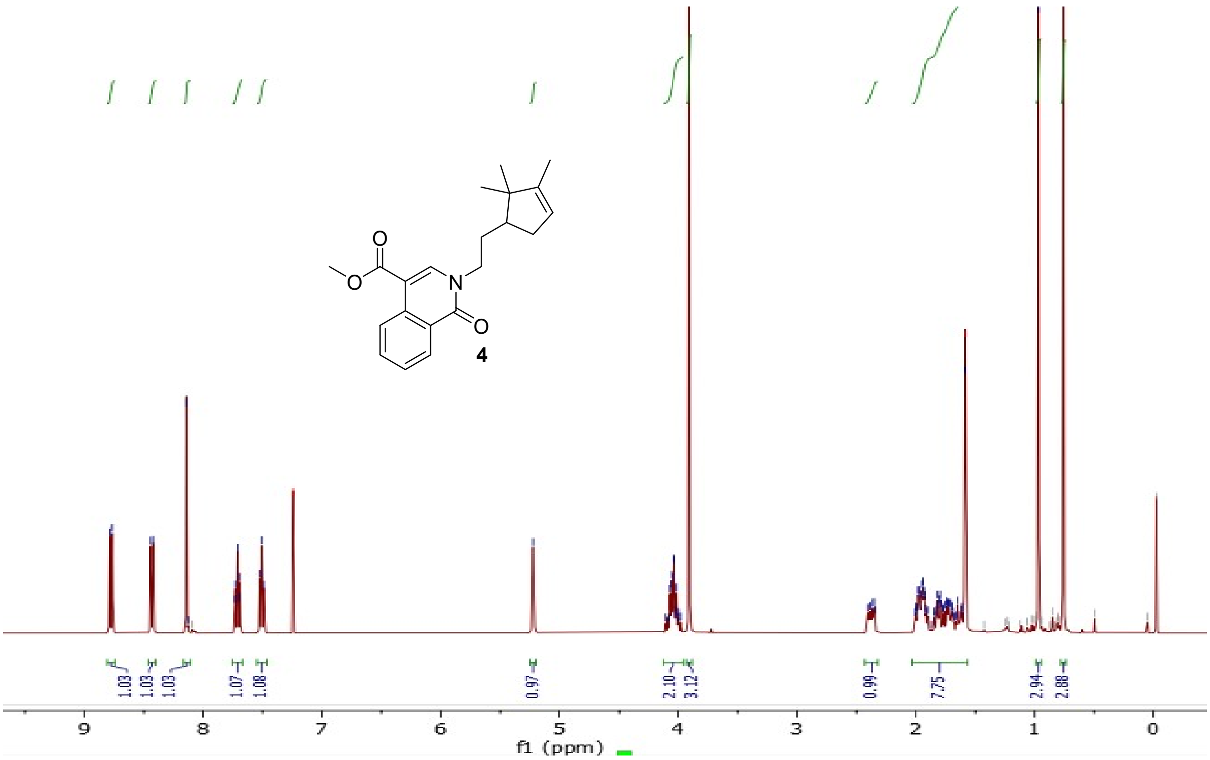

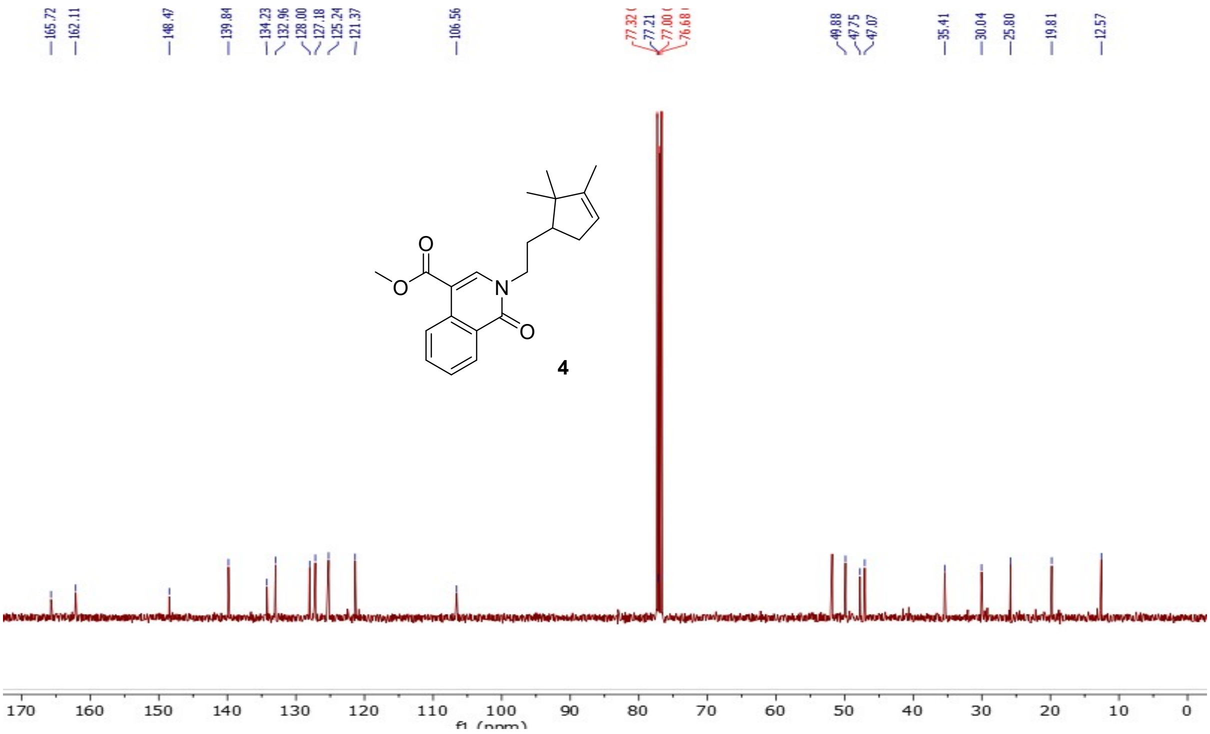

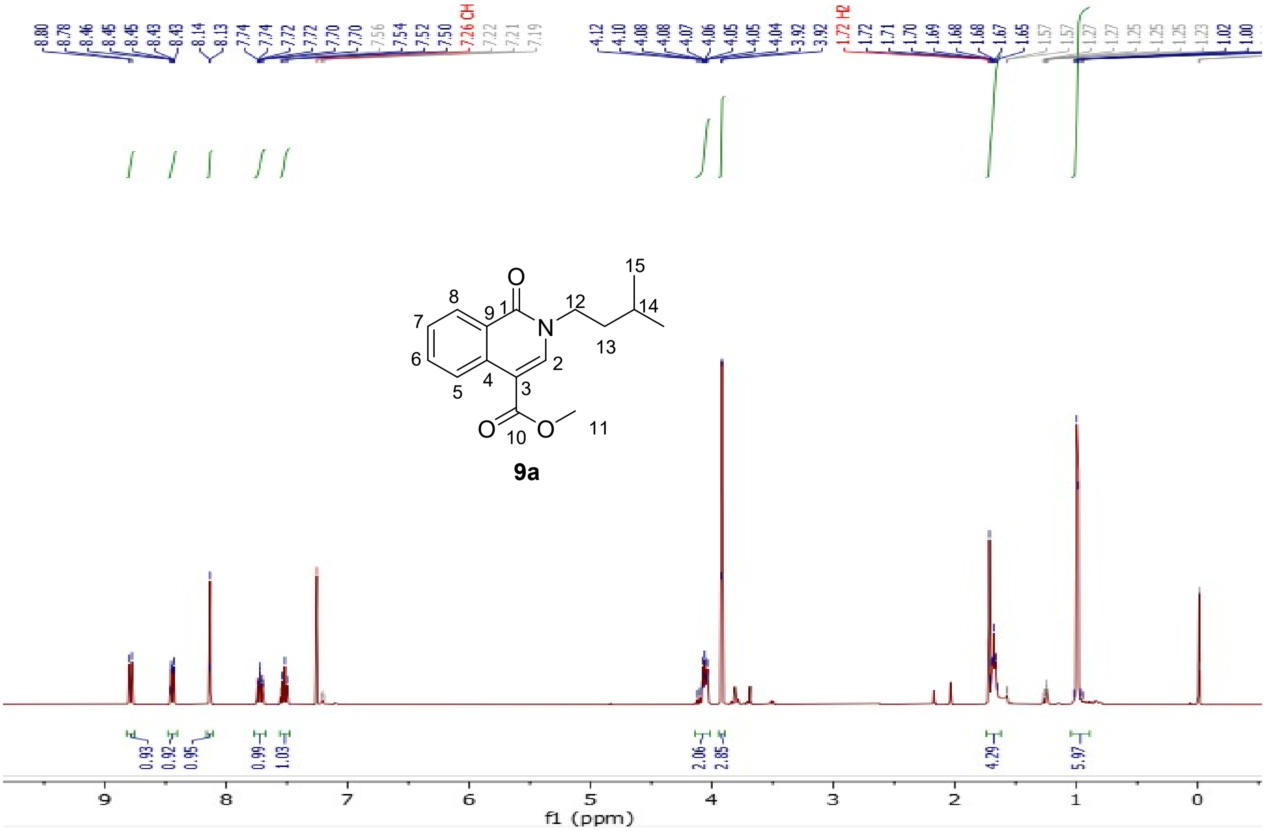
9a
f1 (ppm)

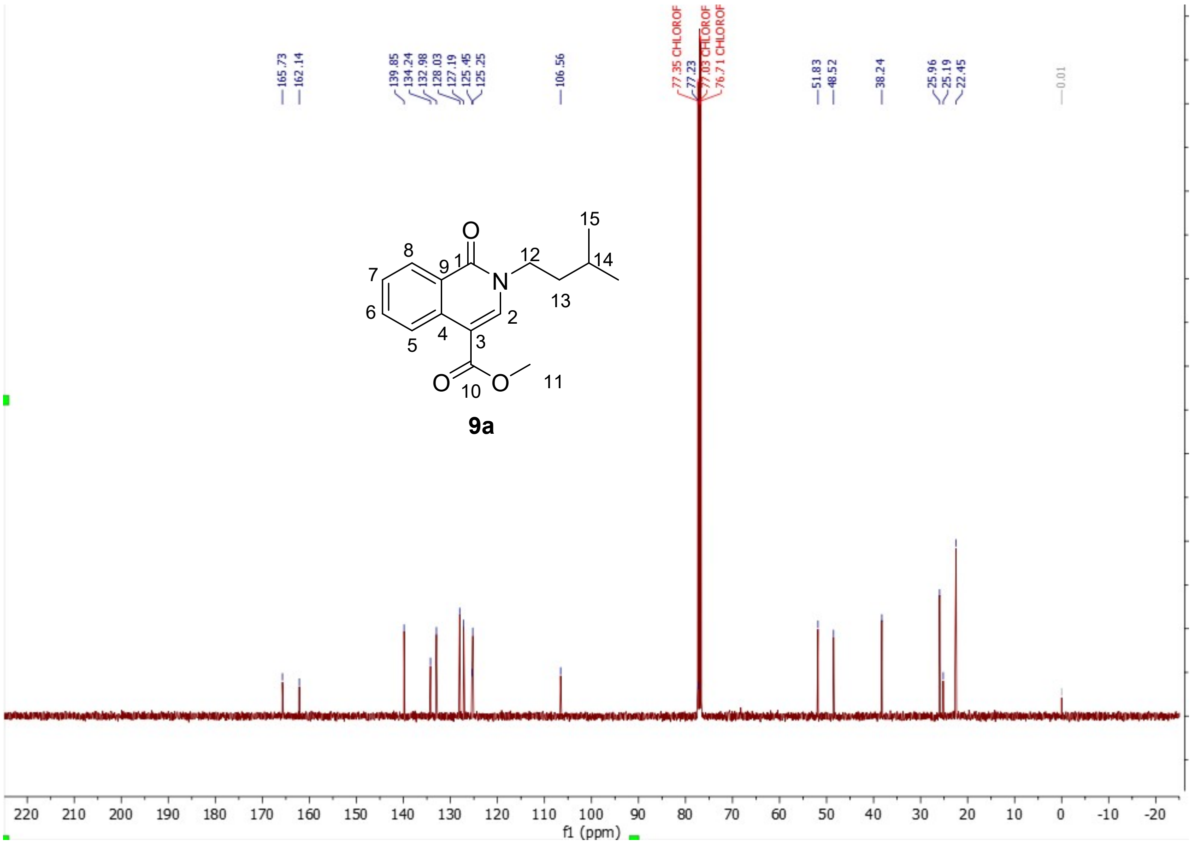
9a
f1 (ppm)

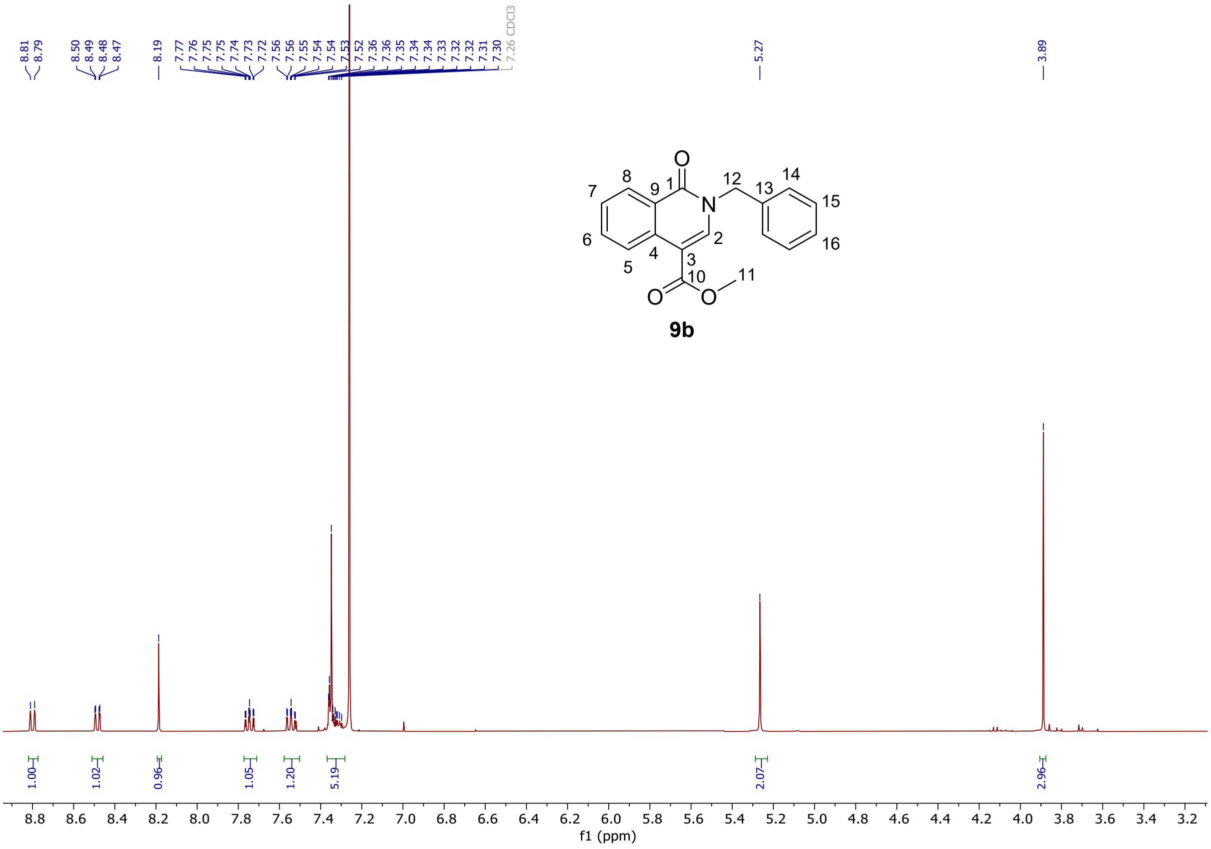
8.81
8.79
8.50
8.49
8.48
8.47
8.19
7.77
7.76
7.75
7.75
7.74
7.73
7.72
7.56
7.56
7.55
7.54
7.54
7.53
7.52
7.36
7.36
7.35
7.34
7.34
7.33
7.32
7.32
7.31
7.30
7.26 CDCl3
5.27
3.89
9b
1.00
1.02
0.96
1.05
1.20
5.19
2.07
2.96
f1 (ppm)

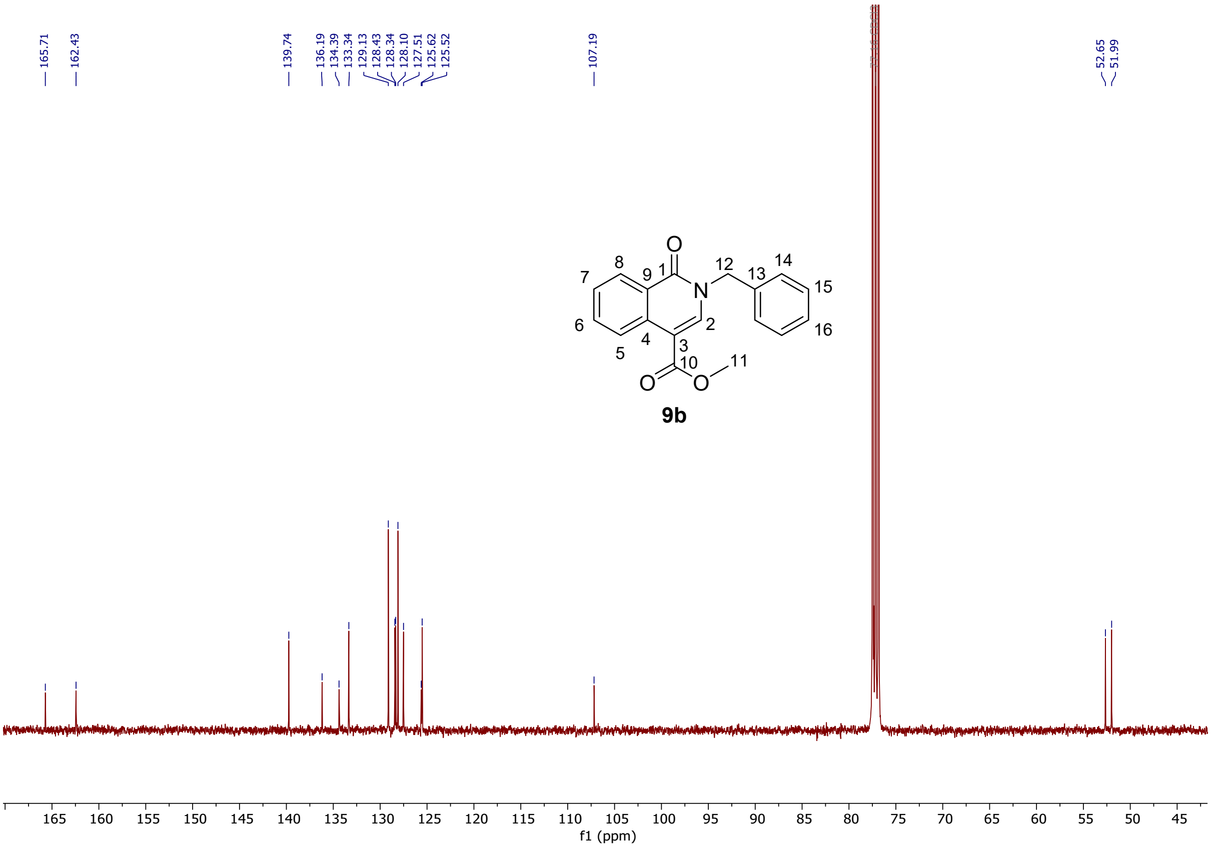
165.71
162.43
139.74
136.19
134.39
133.34
129.13
128.43
128.34
128.10
127.51
125.62
125.52
107.19
52.65
51.99
9b
f1 (ppm)

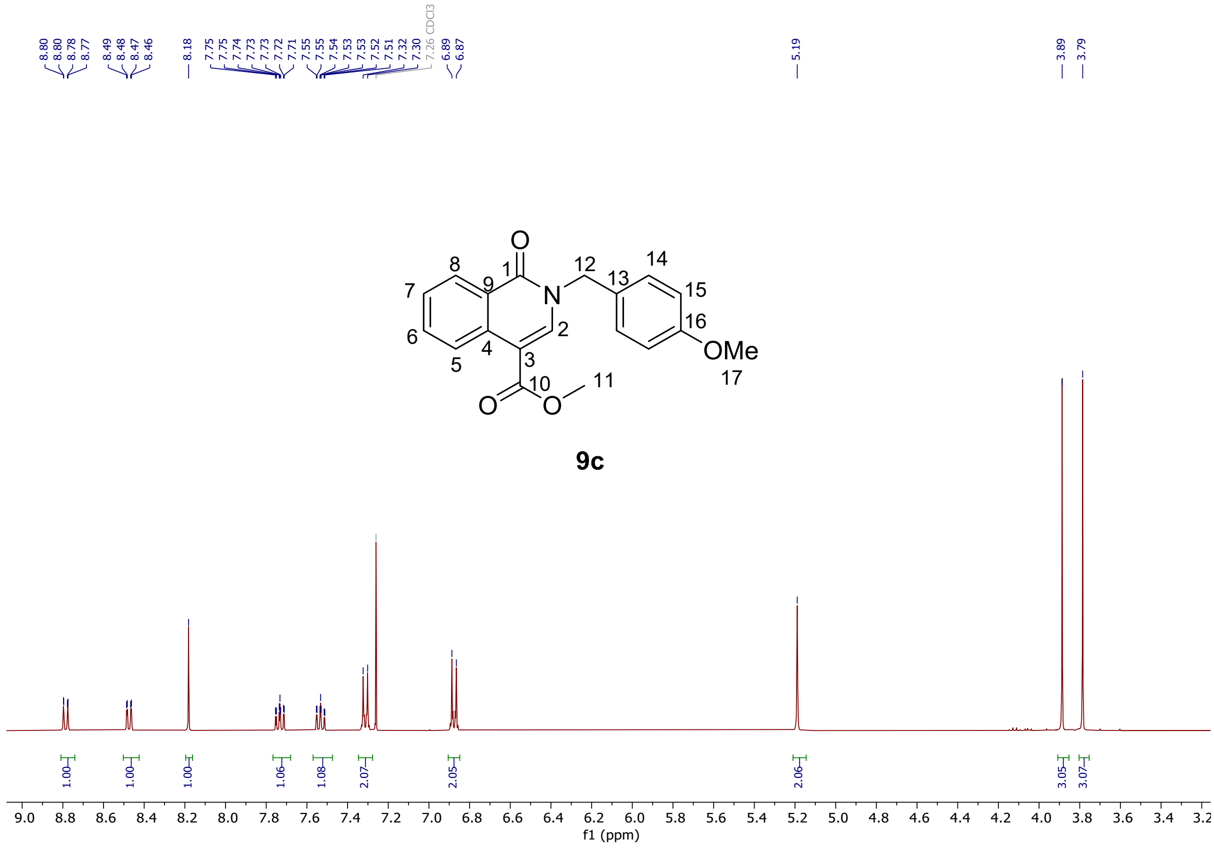
8.80
8.80
8.78
8.77
8.49
8.48
8.47
8.46
8.18
7.75
7.75
7.74
7.73
7.73
7.72
7.71
7.55
7.55
7.54
7.53
7.53
7.52
7.51
7.32
7.30
7.26 CDCl3
6.89
6.87
5.19
3.89
3.79
9c
1.00
1.00
1.00
1.06
1.08
2.07
2.05
2.06
3.05
3.07
f1 (ppm)

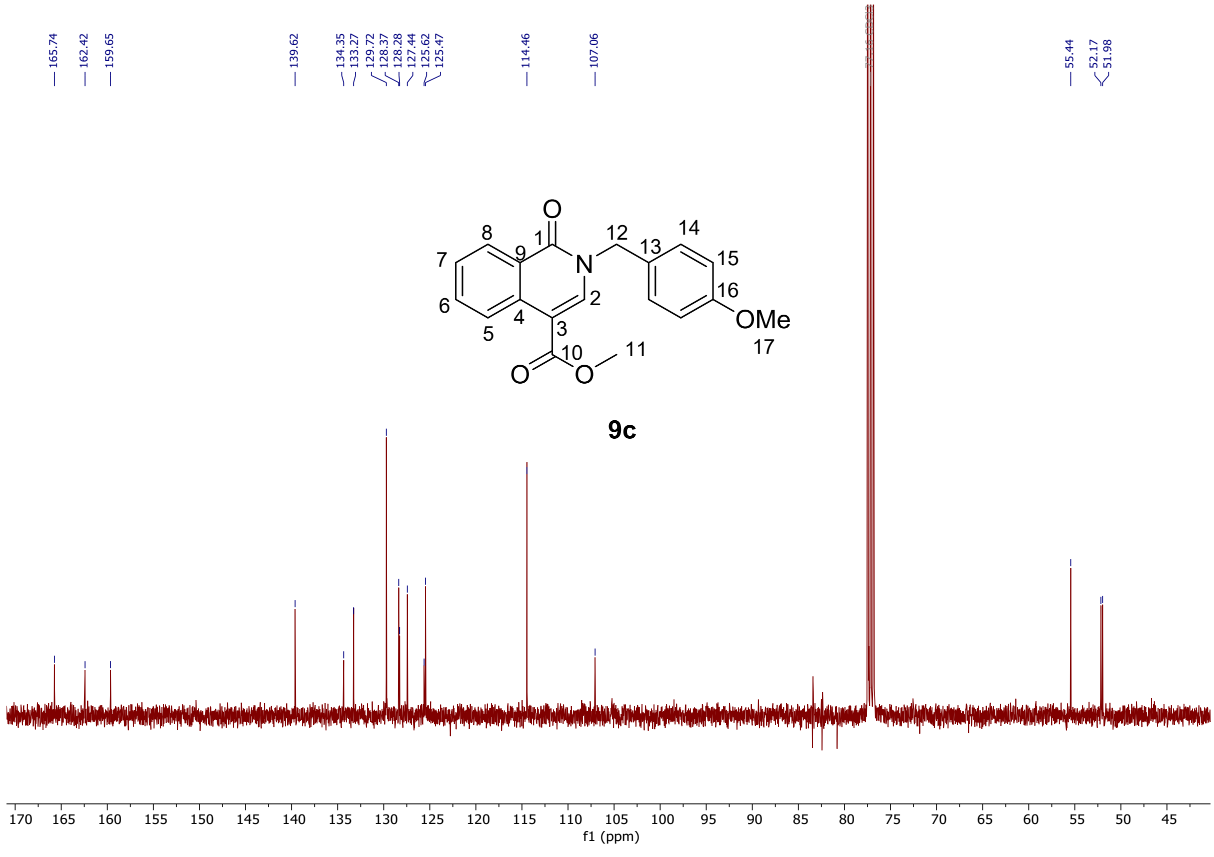
165.74
162.42
159.65
139.62
134.35
133.27
129.72
128.37
128.28
127.44
125.62
125.47
114.46
107.06
55.44
52.17
51.98
9c
f1 (ppm)

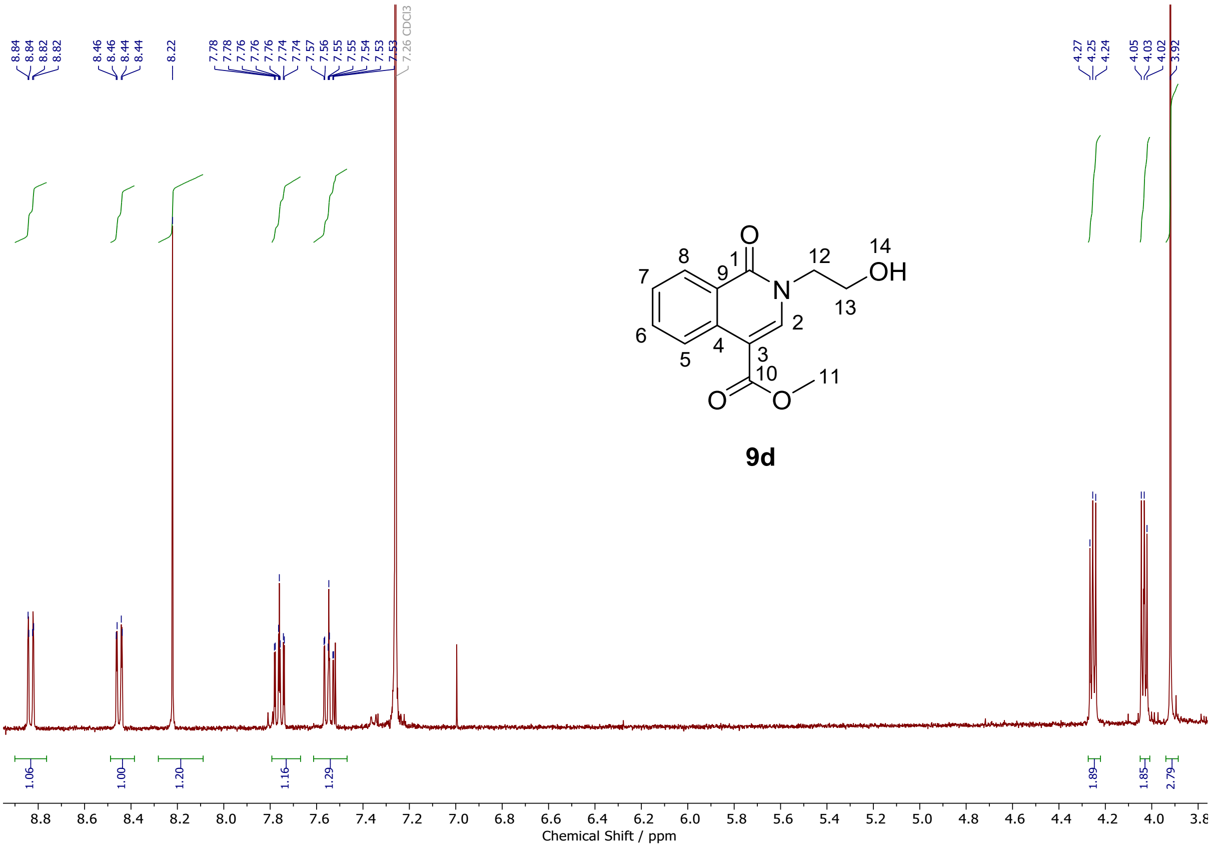
8.84 8.84 8.82 8.82
8.46 8.46 8.44 8.44
8.22
7.78 7.78 7.76 7.76 7.76 7.74 7.74
7.57 7.56 7.55 7.55 7.54 7.53 7.53
7.26 CDCl3
4.27 4.25 4.24
4.05 4.03 4.02
3.92
9d
1.06 1.00 1.20 1.16 1.29 1.89 1.85 2.79
Chemical Shift / ppm

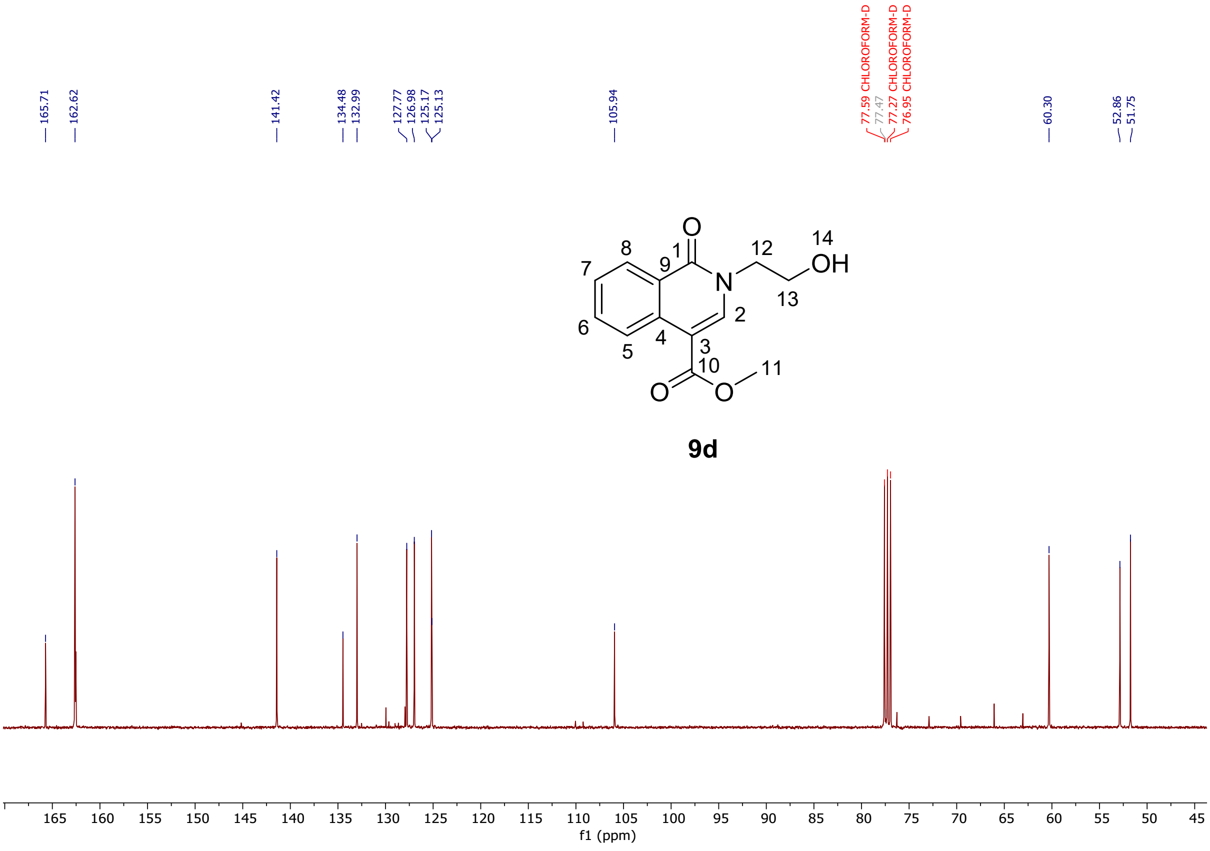
165.71
162.62
141.42
134.48 132.99
127.77 126.98 125.17 125.13
105.94
77.59 CHLOROFORM-D
77.47
77.27 CHLOROFORM-D
76.95 CHLOROFORM-D
60.30
52.86 51.75
9d
f1 (ppm)

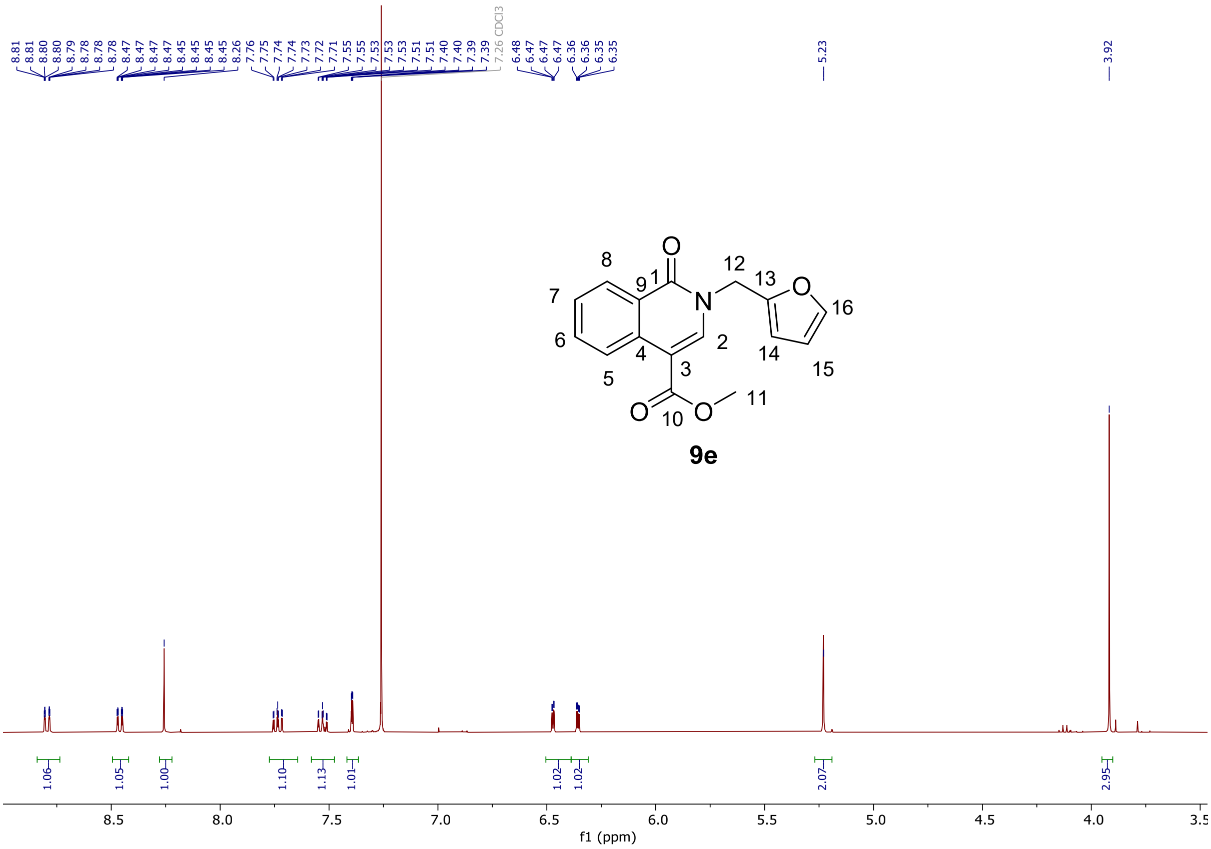
8.81 8.81 8.80 8.79 8.78 8.78 8.78 8.47 8.47 8.47 8.47 8.45 8.45 8.45 8.45 8.26 7.76 7.75 7.74 7.74 7.73 7.72 7.71 7.55 7.55 7.53 7.53 7.53 7.53 7.51 7.51 7.40 7.40 7.39 7.39
7.26 CDCl3
6.48 6.47 6.47 6.47 6.36 6.36 6.35 6.35
5.23
3.92
1.06 1.05 1.00 1.10 1.13 1.01 1.02 1.02 2.07 2.95
8.5 8.0 7.5 7.0 6.5 6.0 5.5 5.0 4.5 4.0 3.5
f1 (ppm)
O 8 9 1 12 13 O 7 N 16 6 4 3 2 14 15 5 O 10 O 11
9e

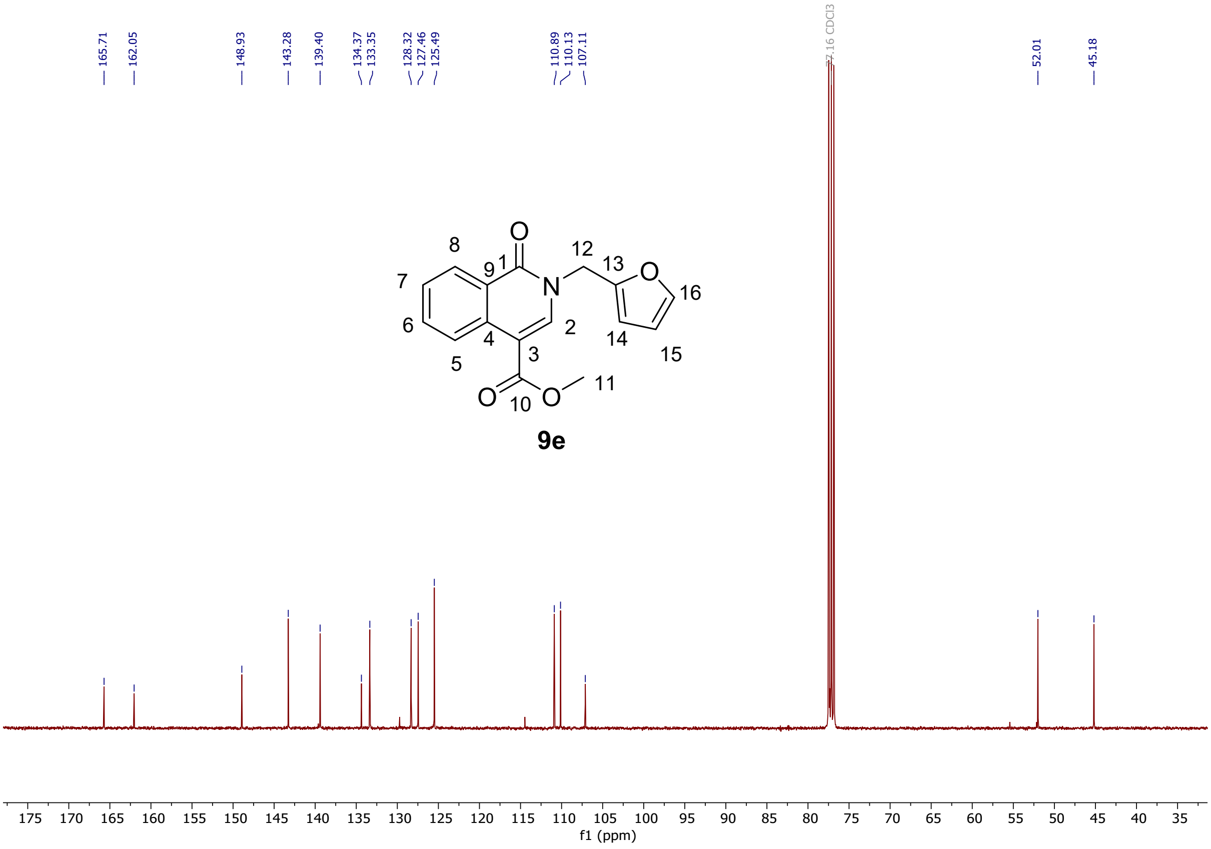
165.71 162.05 148.93 143.28 139.40 134.37 133.35 128.32 127.46 125.49 110.89 110.13 107.11
77.16 CDCl3
52.01 45.18
175 170 165 160 155 150 145 140 135 130 125 120 115 110 105 100 95 90 85 80 75 70 65 60 55 50 45 40 35
f1 (ppm)
O 8 9 1 12 13 O 7 N 16 6 4 3 2 14 15 5 O 10 O 11
9e

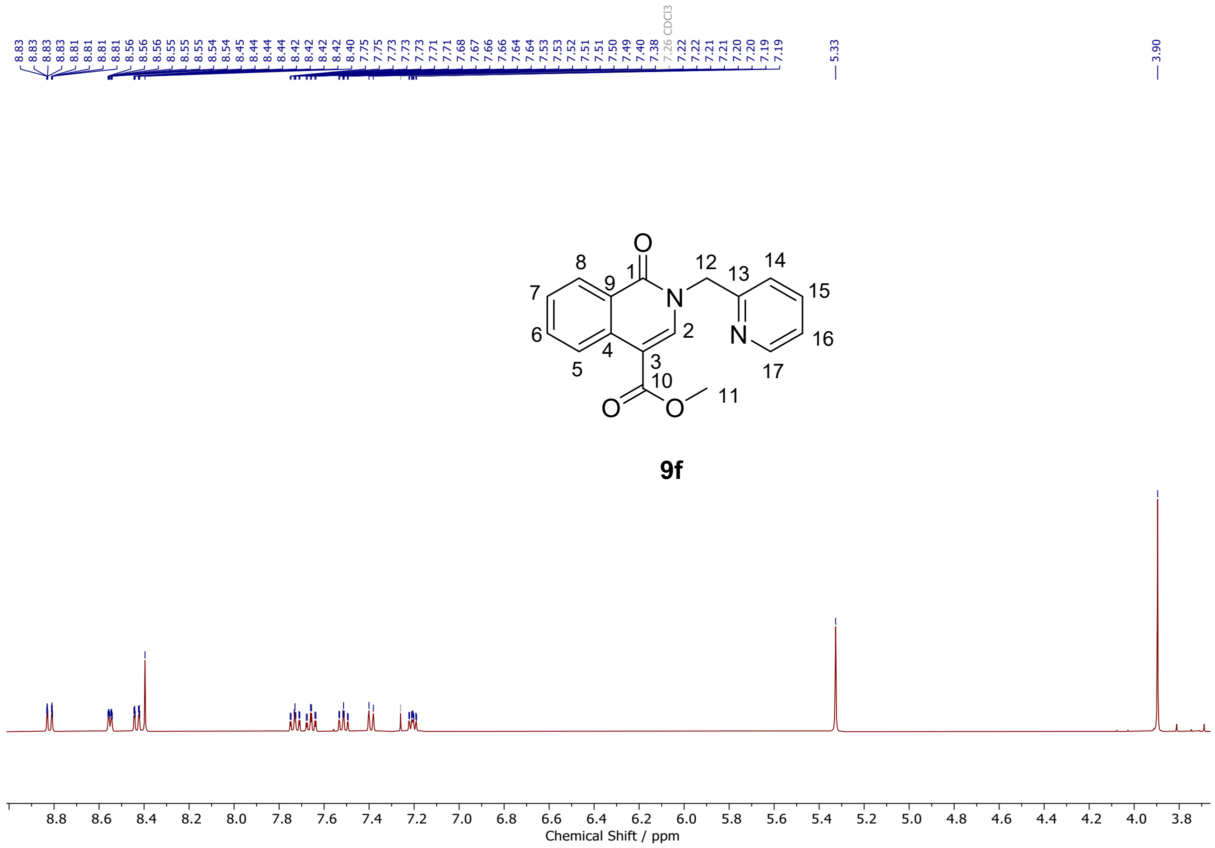
8.83 8.83 8.83 8.83 8.81 8.81 8.81 8.81 8.56 8.56 8.56 8.55 8.55 8.55 8.54 8.54 8.45 8.44 8.44 8.42 8.42 8.42 8.42 8.40 7.75 7.75 7.73 7.73 7.73 7.71 7.71 7.68 7.67 7.66 7.66 7.64 7.53 7.53 7.52 7.51 7.51 7.50 7.49 7.40 7.38 7.26 CDCl3 7.22 7.22 7.21 7.21 7.20 7.20 7.19 7.19
5.33
3.90
9f
8.8 8.6 8.4 8.2 8.0 7.8 7.6 7.4 7.2 7.0 6.8 6.6 6.4 6.2 6.0 5.8 5.6 5.4 5.2 5.0 4.8 4.6 4.4 4.2 4.0 3.8
Chemical Shift / ppm

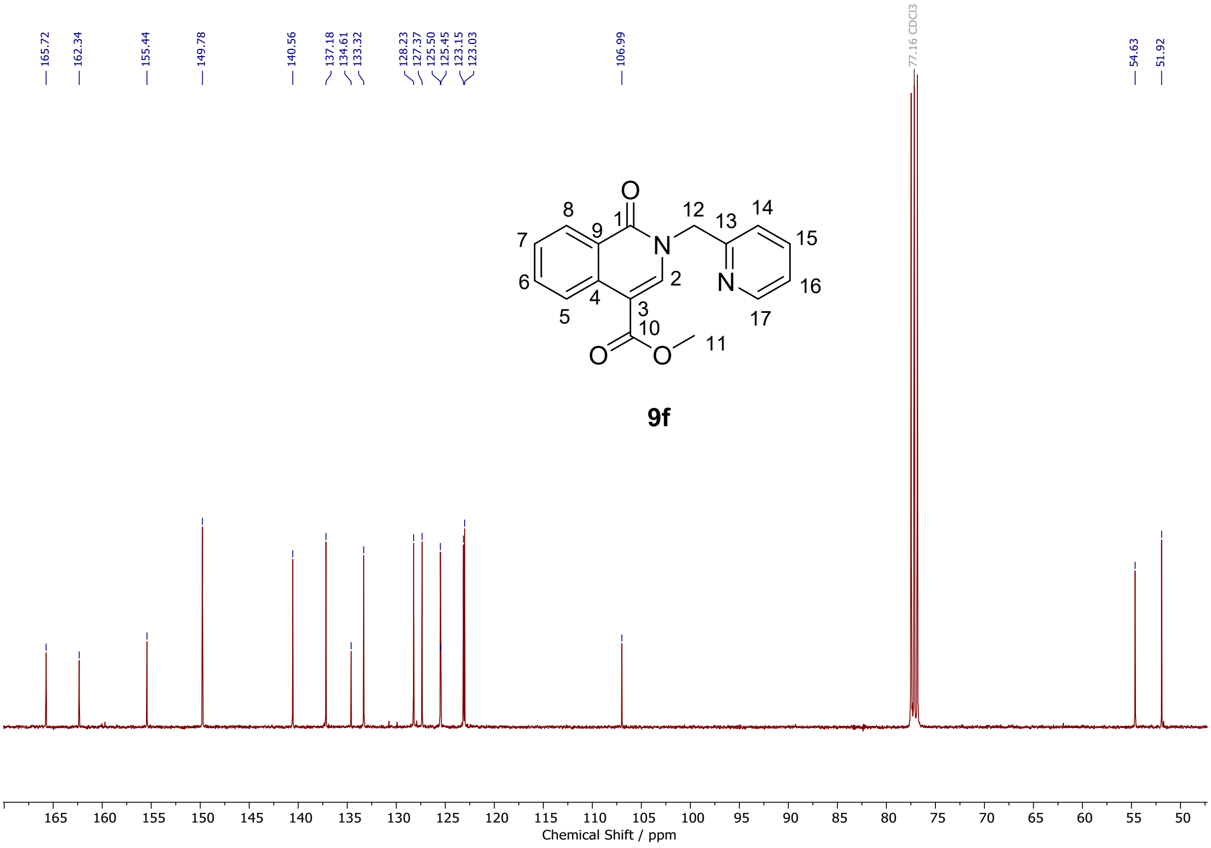
165.72
162.34
155.44
149.78
140.56
137.18
134.61
133.32
128.23
127.37
125.50
125.45
123.15
123.03
106.99
77.16 CDCl3
54.63
51.92
9f
Chemical Shift / ppm

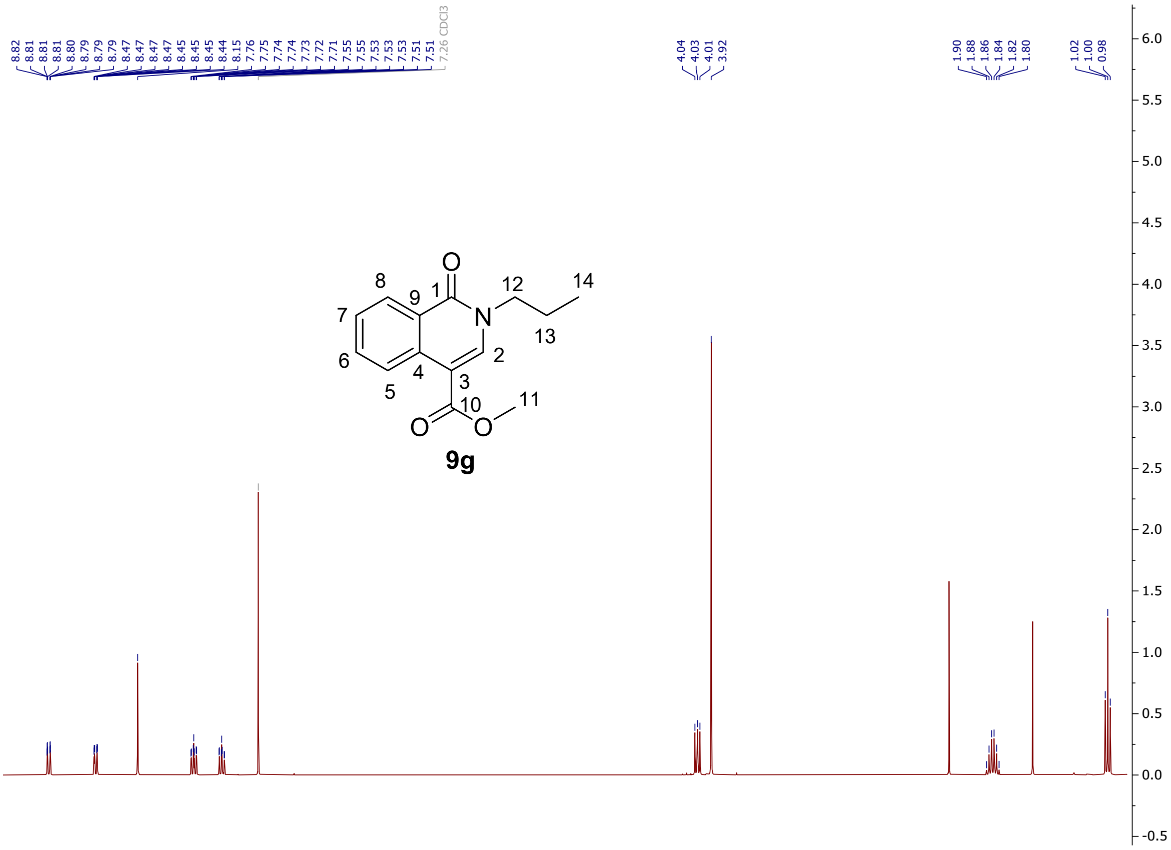
8.82
8.81
8.81
8.81
8.80
8.79
8.79
8.79
8.47
8.47
8.47
8.47
8.45
8.45
8.45
8.44
8.15
7.76
7.75
7.74
7.74
7.73
7.72
7.71
7.55
7.55
7.53
7.53
7.53
7.51
7.51
7.26 CDCl3
4.04
4.03
4.01
3.92
1.90
1.88
1.86
1.84
1.82
1.80
1.02
1.00
0.98
9g

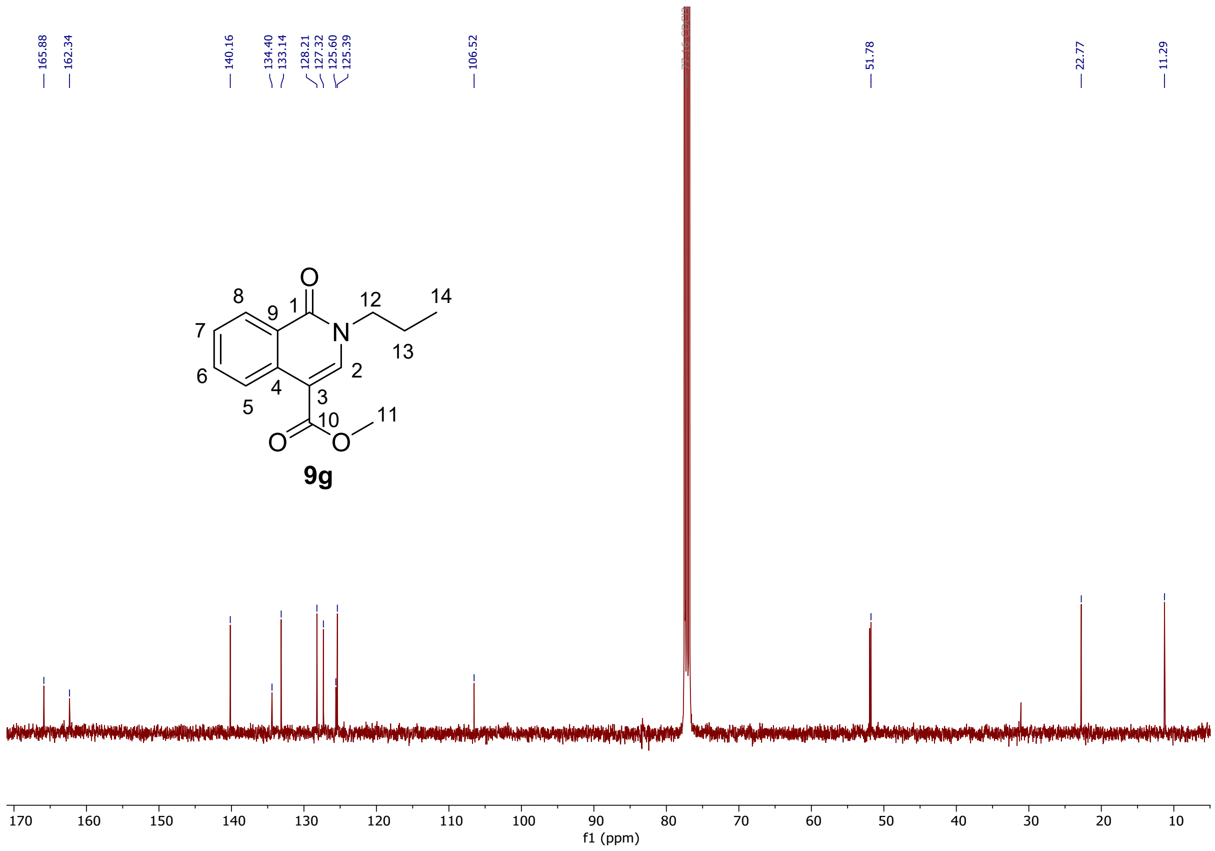
165.88
162.34
140.16
134.40
133.14
128.21
127.32
125.60
125.39
106.52
51.78
22.77
11.29
O
N
O
O
1
2
3
4
5
6
7
8
9
10
11
12
13
14
9g
170
160
150
140
130
120
110
100
90
80
70
60
50
40
30
20
10
f1 (ppm)

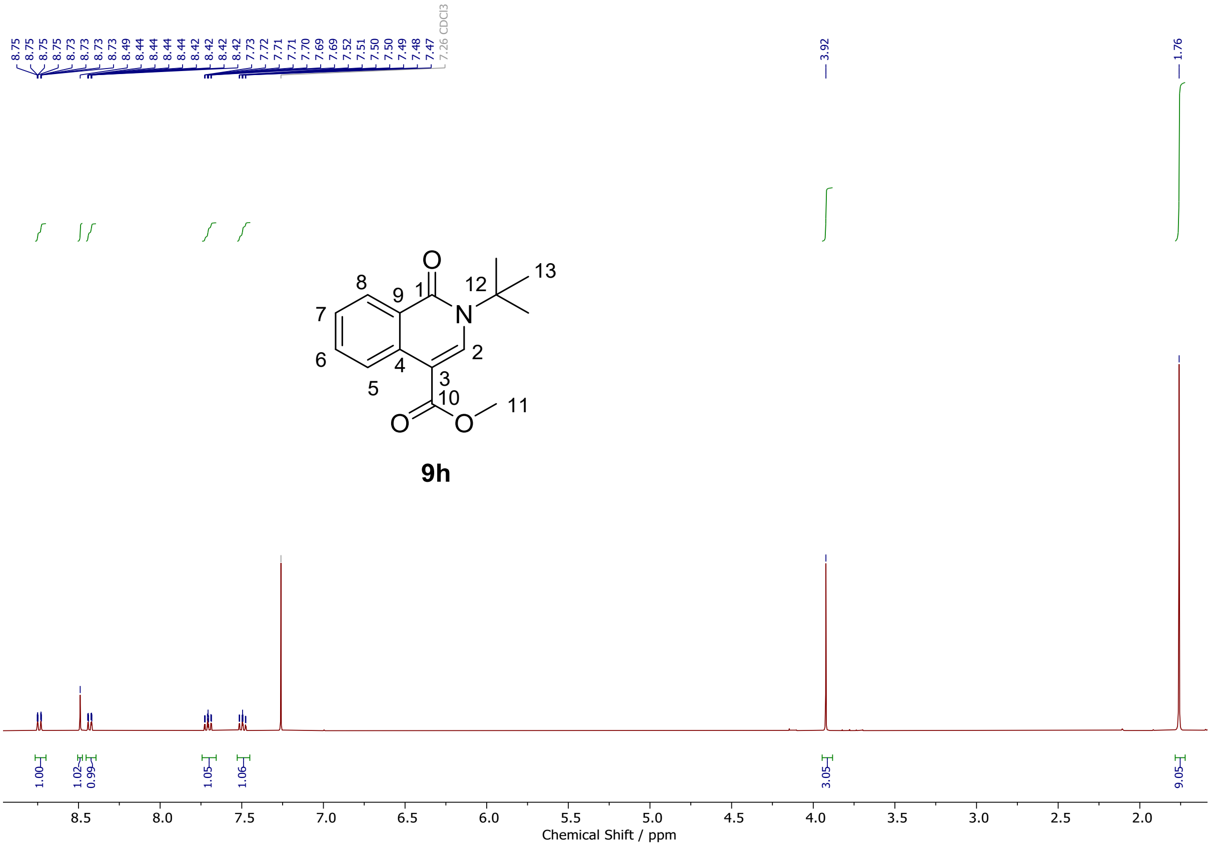
8.75
8.75
8.75
8.75
8.73
8.73
8.73
8.73
8.49
8.44
8.44
8.44
8.44
8.42
8.42
8.42
8.42
7.73
7.72
7.71
7.71
7.70
7.69
7.69
7.52
7.51
7.50
7.50
7.49
7.48
7.47
7.26 CDCl3
3.92
1.76
O
N
8
9
1
12
13
7
6
5
4
3
2
10
11
O
O
9h
1.00
1.02
0.99
1.05
1.06
3.05
9.05
Chemical Shift / ppm

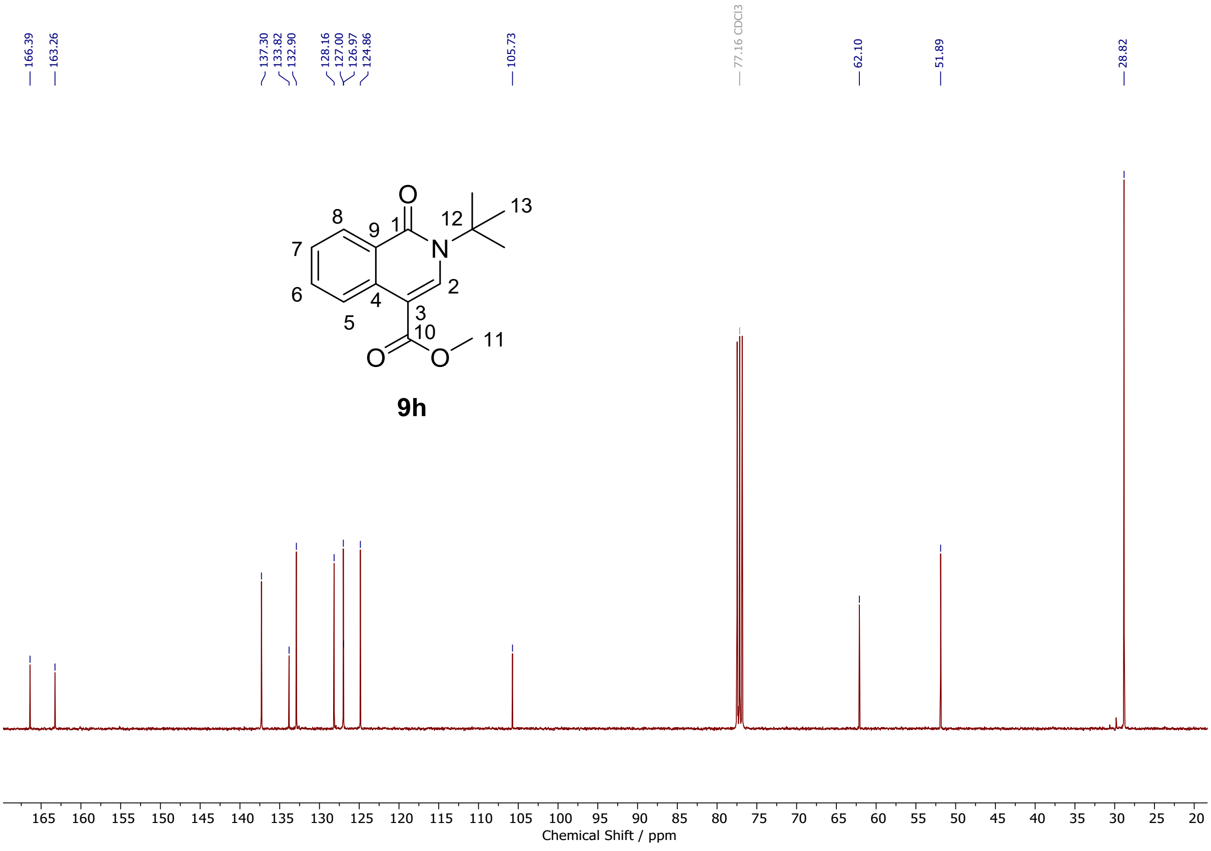
166.39
163.26
137.30
133.82
132.90
128.16
127.00
126.97
124.86
105.73
77.16 CDCl3
62.10
51.89
28.82
O
N
8
9
1
12
13
7
6
5
4
3
2
10
11
O
O
9h
Chemical Shift / ppm

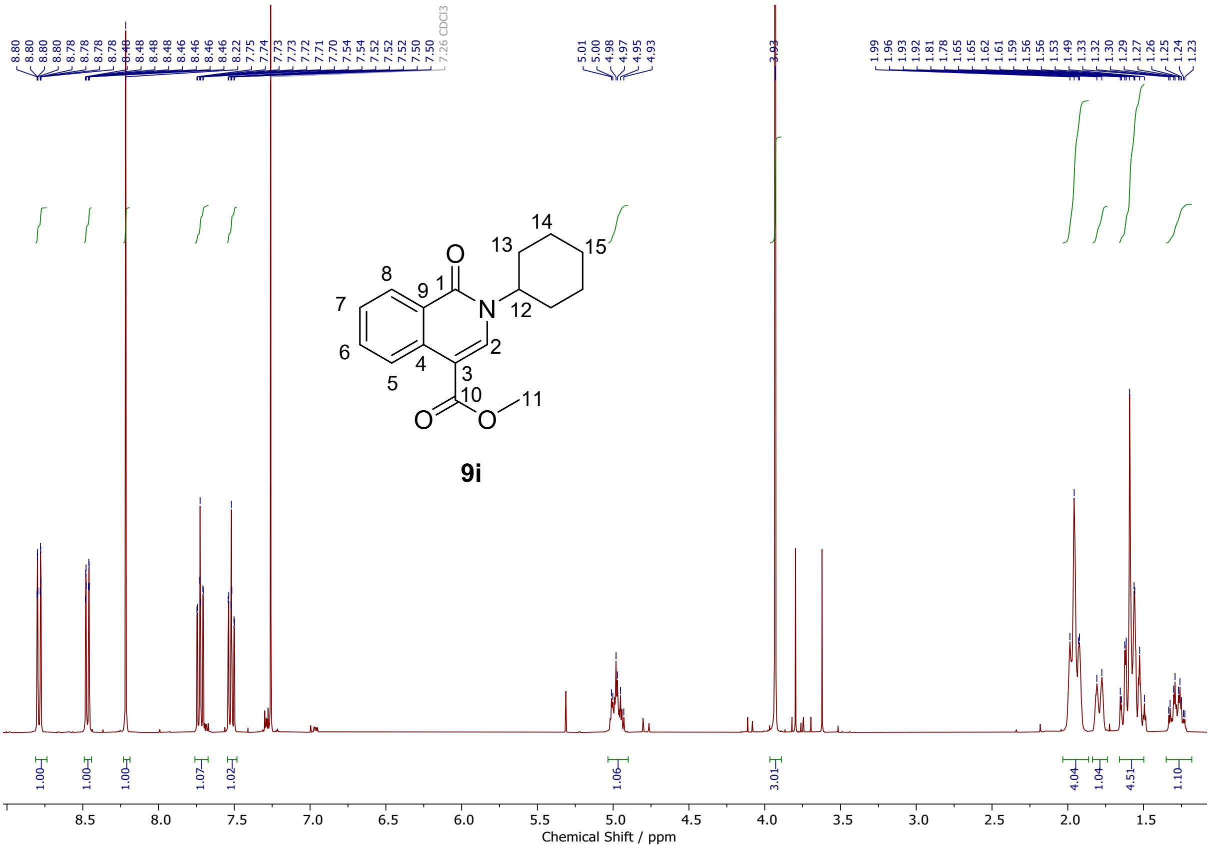
8.80 8.80 8.80 8.80 8.78 8.78 8.78 8.48 8.48 8.48 8.46 8.46 8.46 8.46 8.22 7.75 7.74 7.73 7.73 7.72 7.71 7.70 7.54 7.54 7.52 7.52 7.52 7.50 7.50
7.26 CDCl3
5.01 5.00 4.98 4.97 4.95 4.93
3.93
1.99 1.96 1.93 1.92 1.81 1.78 1.65 1.65 1.62 1.61 1.59 1.56 1.53 1.49 1.33 1.32 1.30 1.29 1.27 1.26 1.25 1.24 1.23
1.00 1.00 1.00 1.07 1.02 1.06 3.01 4.04 1.04 4.51 1.10
9i
Chemical Shift / ppm

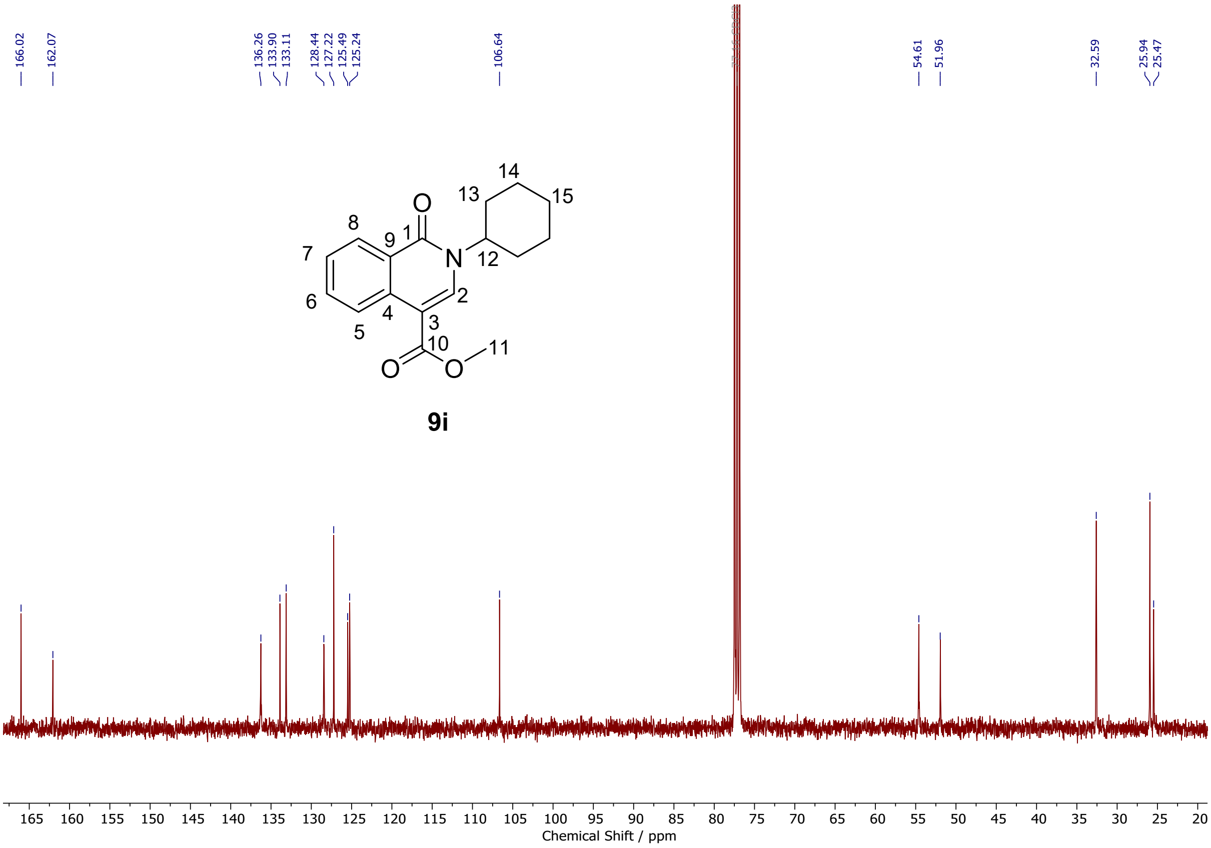
166.02
162.07
136.26 133.90 133.11
128.44 127.22 125.49 125.24
106.64
54.61 51.96
32.59
25.94 25.47
9i
Chemical Shift / ppm

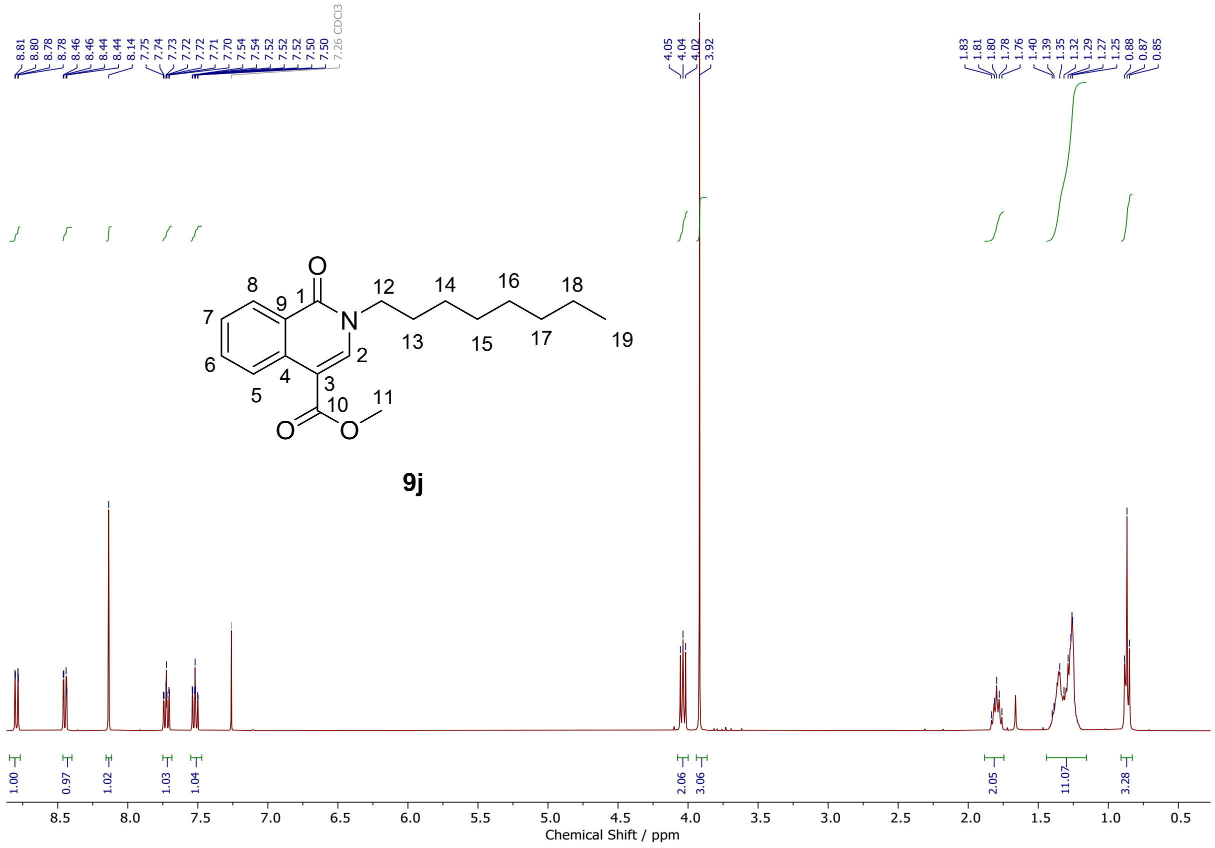
8.81
8.80
8.78
8.78
8.46
8.46
8.44
8.44
8.14
7.75
7.74
7.73
7.72
7.72
7.71
7.70
7.54
7.54
7.52
7.52
7.52
7.50
7.50
7.26 CDCl3
4.05
4.04
4.02
3.92
1.83
1.81
1.80
1.78
1.76
1.40
1.39
1.35
1.32
1.29
1.27
1.25
0.88
0.87
0.85
O
N
1
2
3
4
5
6
7
8
9
10
11
12
13
14
15
16
17
18
19
O
O
9j
1.00
0.97
1.02
1.03
1.04
2.06
3.06
2.05
11.07
3.28
Chemical Shift / ppm

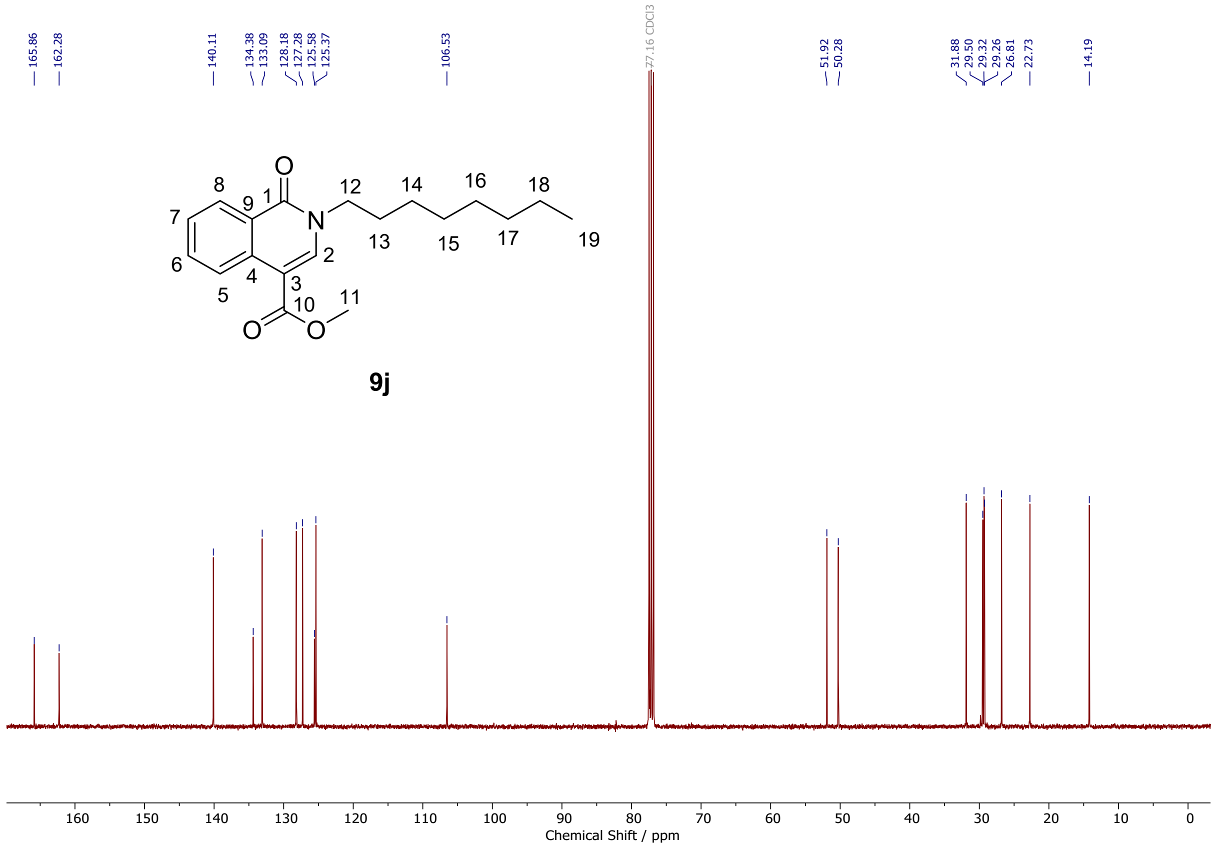
165.86
162.28
140.11
134.38
133.09
128.18
127.28
125.58
125.37
106.53
77.16 CDCl3
51.92
50.28
31.88
29.50
29.32
29.26
26.81
22.73
14.19
9j
Chemical Shift / ppm

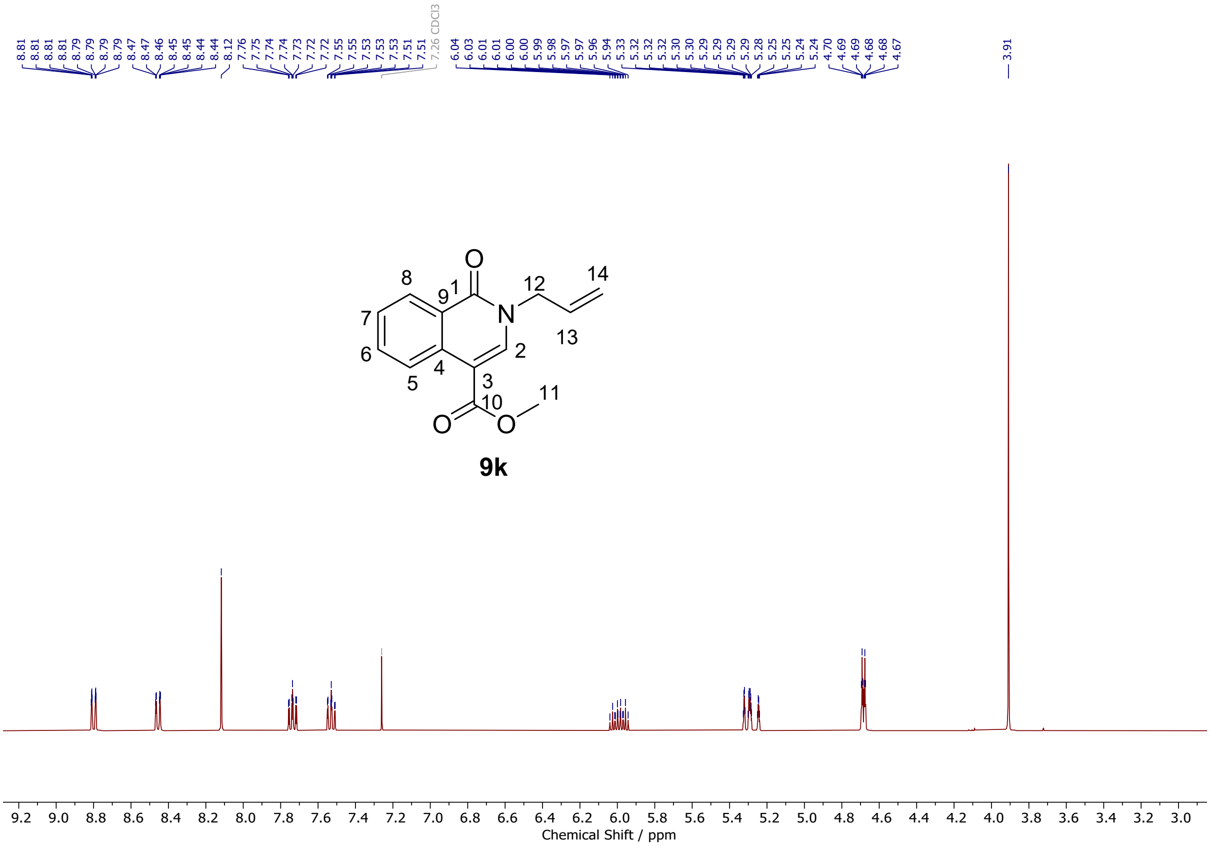
8.81 8.81 8.81 8.81 8.79 8.79 8.79 8.79 8.47 8.46 8.45 8.45 8.45 8.44 8.12 7.76 7.75 7.74 7.74 7.73 7.72 7.72 7.55 7.55 7.53 7.53 7.53 7.51 7.26 CDCl3 6.04 6.03 6.01 6.01 6.00 6.00 5.99 5.98 5.97 5.97 5.96 5.94 5.33 5.32 5.32 5.32 5.30 5.29 5.29 5.29 5.29 5.28 5.25 5.25 5.24 5.24 4.70 4.69 4.69 4.68 4.68 4.67
3.91
9k
9.2 9.0 8.8 8.6 8.4 8.2 8.0 7.8 7.6 7.4 7.2 7.0 6.8 6.6 6.4 6.2 6.0 5.8 5.6 5.4 5.2 5.0 4.8 4.6 4.4 4.2 4.0 3.8 3.6 3.4 3.2 3.0
Chemical Shift / ppm

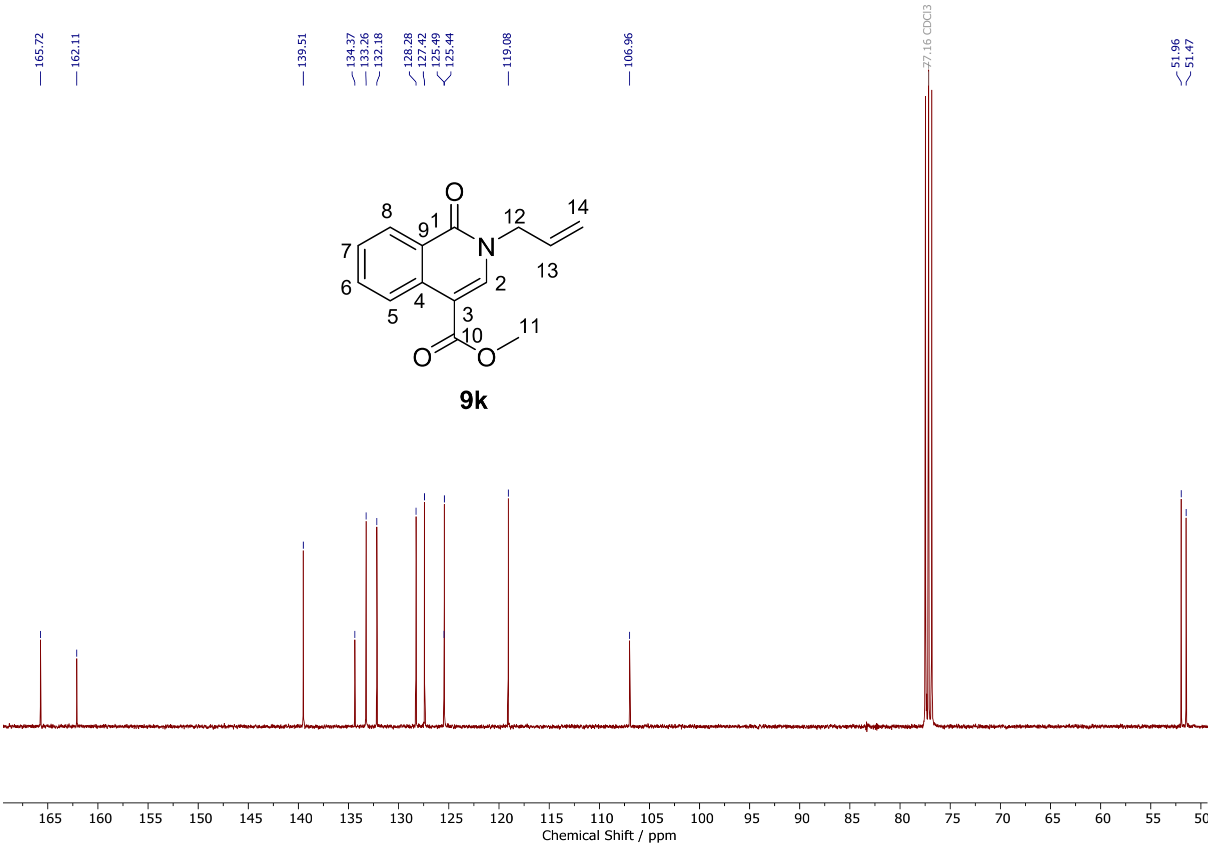
165.72
162.11
139.51
134.37 133.26 132.18
128.28 127.42 125.49 125.44
119.08
106.96
77.16 CDCl3
51.96 51.47
9k
165 160 155 150 145 140 135 130 125 120 115 110 105 100 95 90 85 80 75 70 65 60 55 50
Chemical Shift / ppm

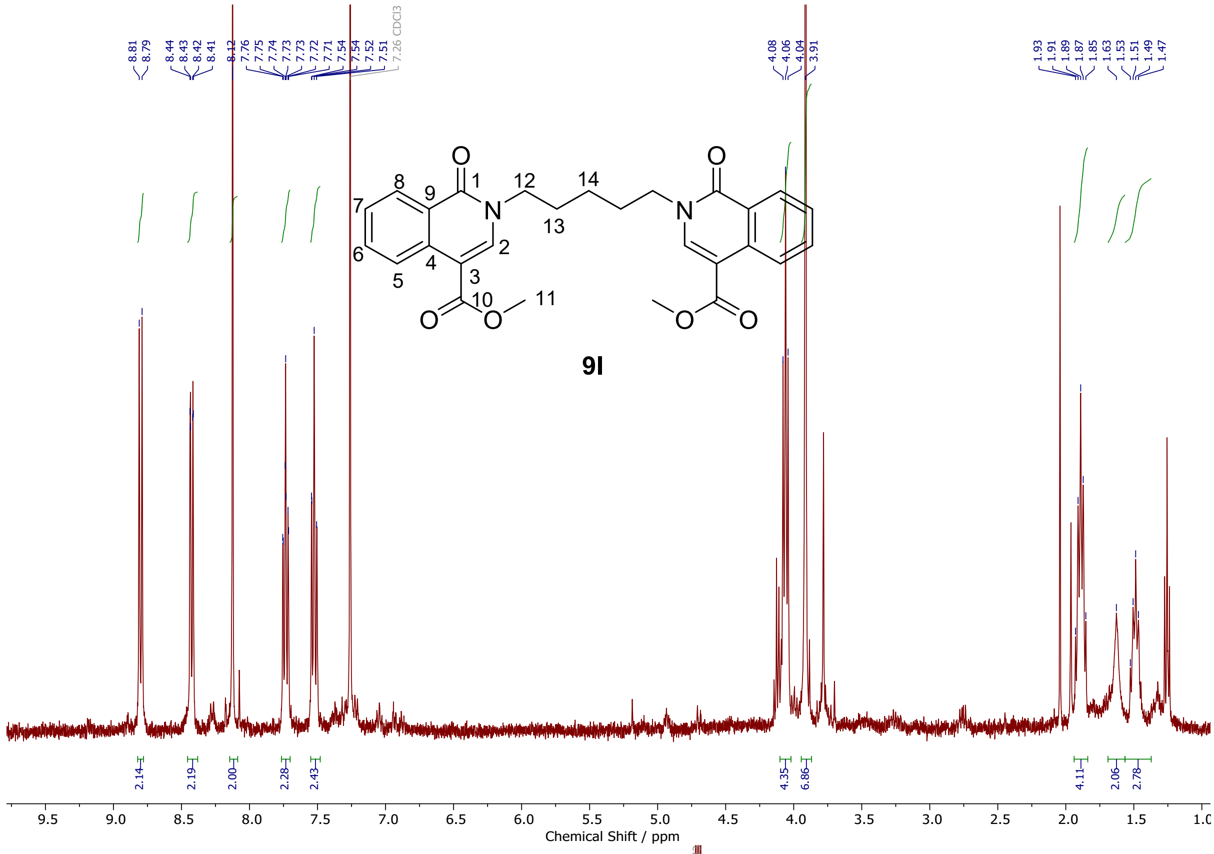
8.81
8.79
8.44
8.43
8.42
8.41
8.12
7.76
7.75
7.74
7.73
7.73
7.72
7.71
7.54
7.54
7.52
7.51
7.26 CDCl3
4.08
4.06
4.04
3.91
1.93
1.91
1.89
1.87
1.85
1.63
1.53
1.51
1.49
1.47
9l
2.14
2.19
2.00
2.28
2.43
4.35
6.86
4.11
2.06
2.78
Chemical Shift / ppm

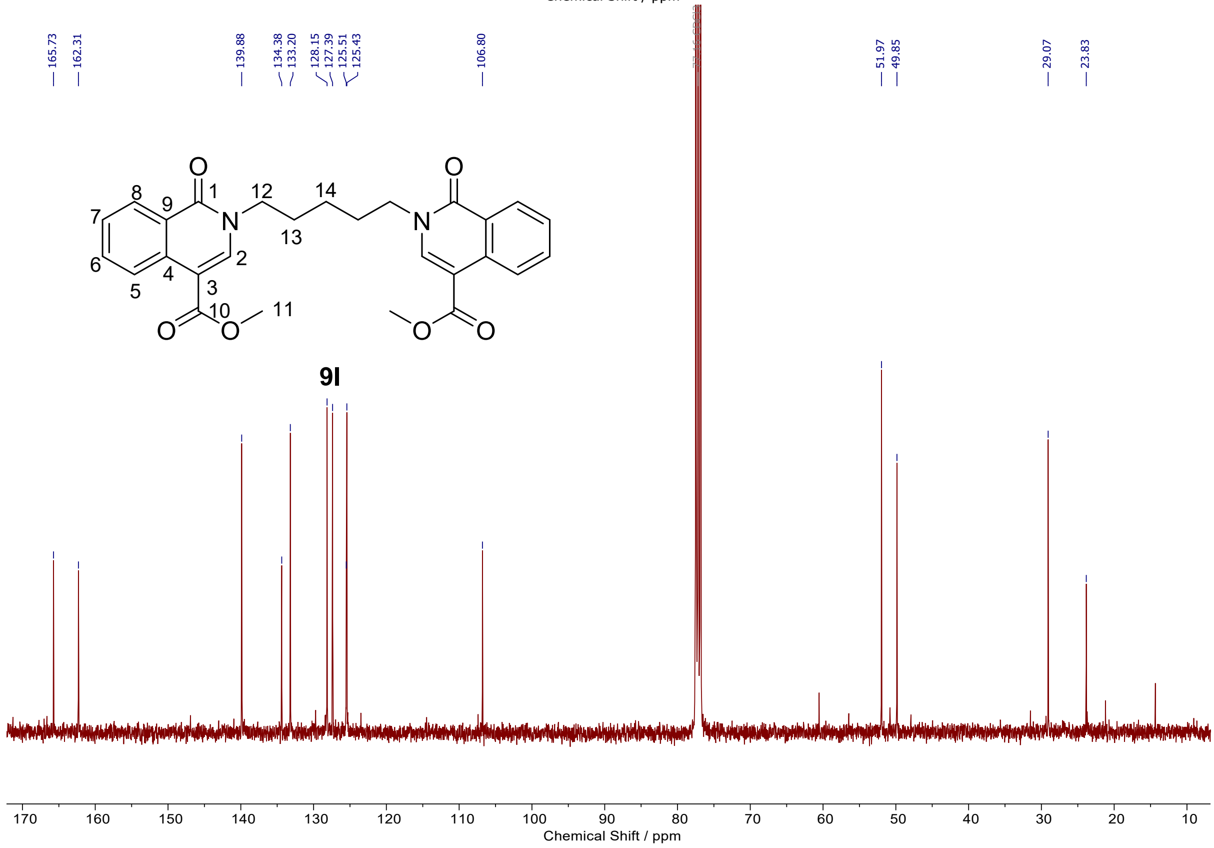
165.73
162.31
139.88
134.38
133.20
128.15
127.39
125.51
125.43
106.80
51.97
49.85
29.07
23.83
9l
Chemical Shift / ppm

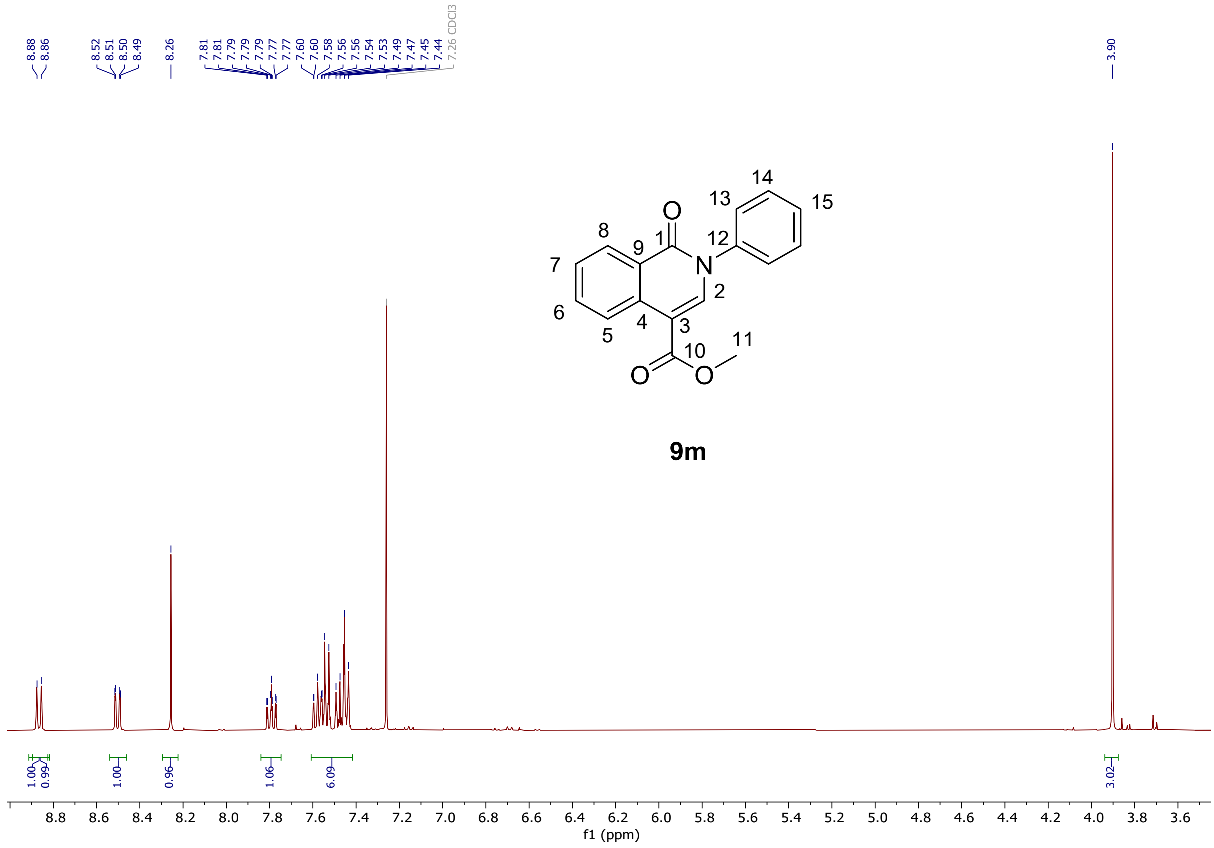
8.88
8.86
8.52
8.51
8.50
8.49
8.26
7.81
7.81
7.79
7.79
7.79
7.77
7.77
7.60
7.60
7.58
7.56
7.56
7.54
7.53
7.49
7.47
7.45
7.44
7.26 CDCl3
3.90
9m
1.00
0.99
1.00
0.96
1.06
6.09
3.02
f1 (ppm)

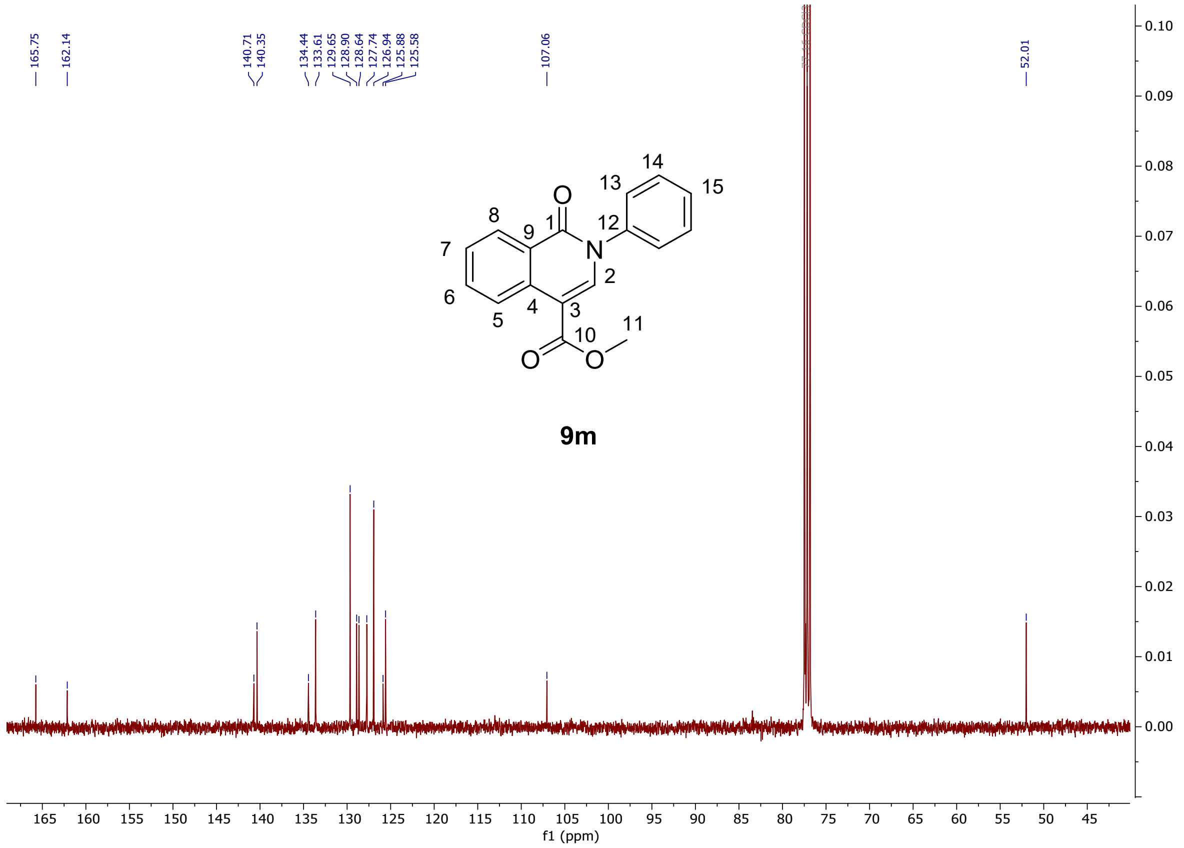
165.75
162.14
140.71
140.35
134.44
133.61
129.65
128.90
128.64
127.74
126.94
125.88
125.58
107.06
52.01
9m
f1 (ppm)

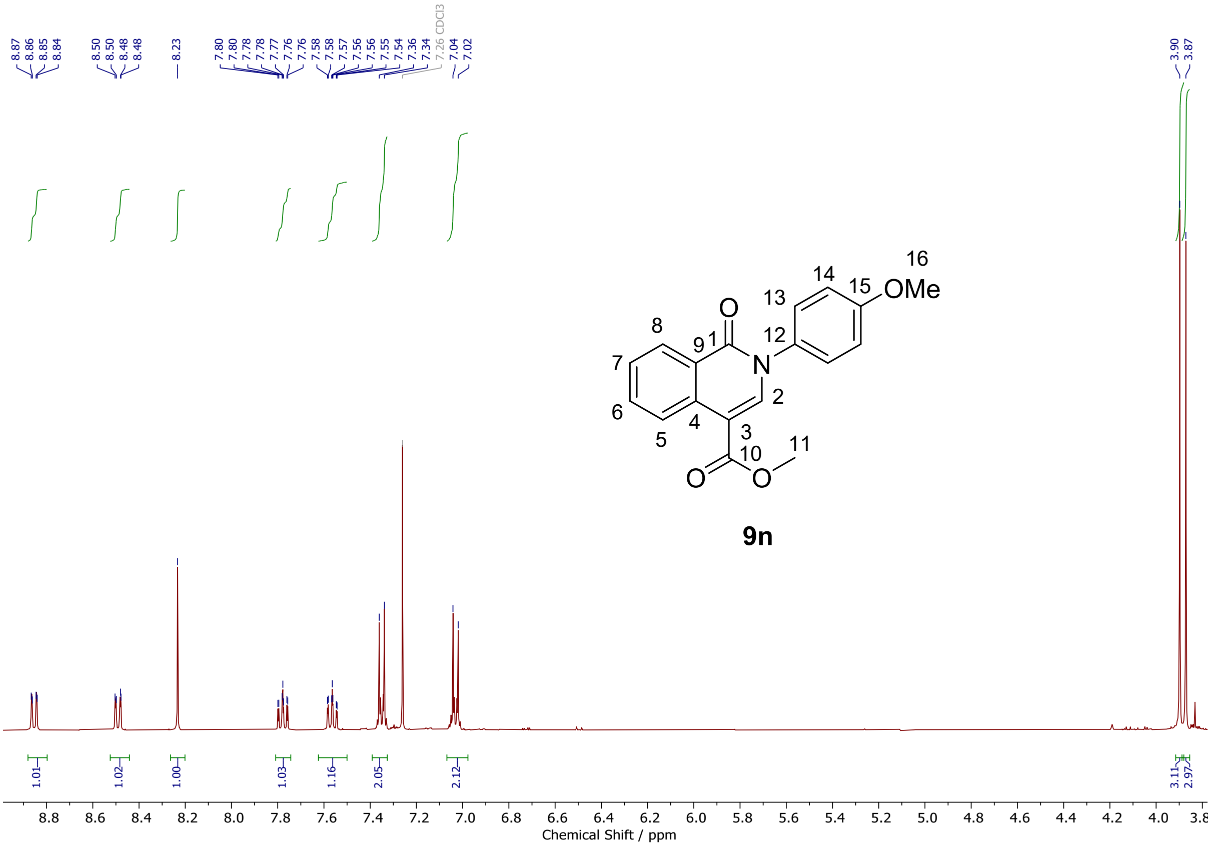

8.87
8.86
8.85
8.84
8.50
8.50
8.48
8.48
8.23
7.80
7.80
7.78
7.77
7.76
7.76
7.58
7.58
7.57
7.56
7.56
7.55
7.54
7.36
7.34
7.26 CDCl3
7.04
7.02
3.90
3.87
9n
1.01
1.02
1.00
1.03
1.16
2.05
2.12
3.11
2.97
Chemical Shift / ppm

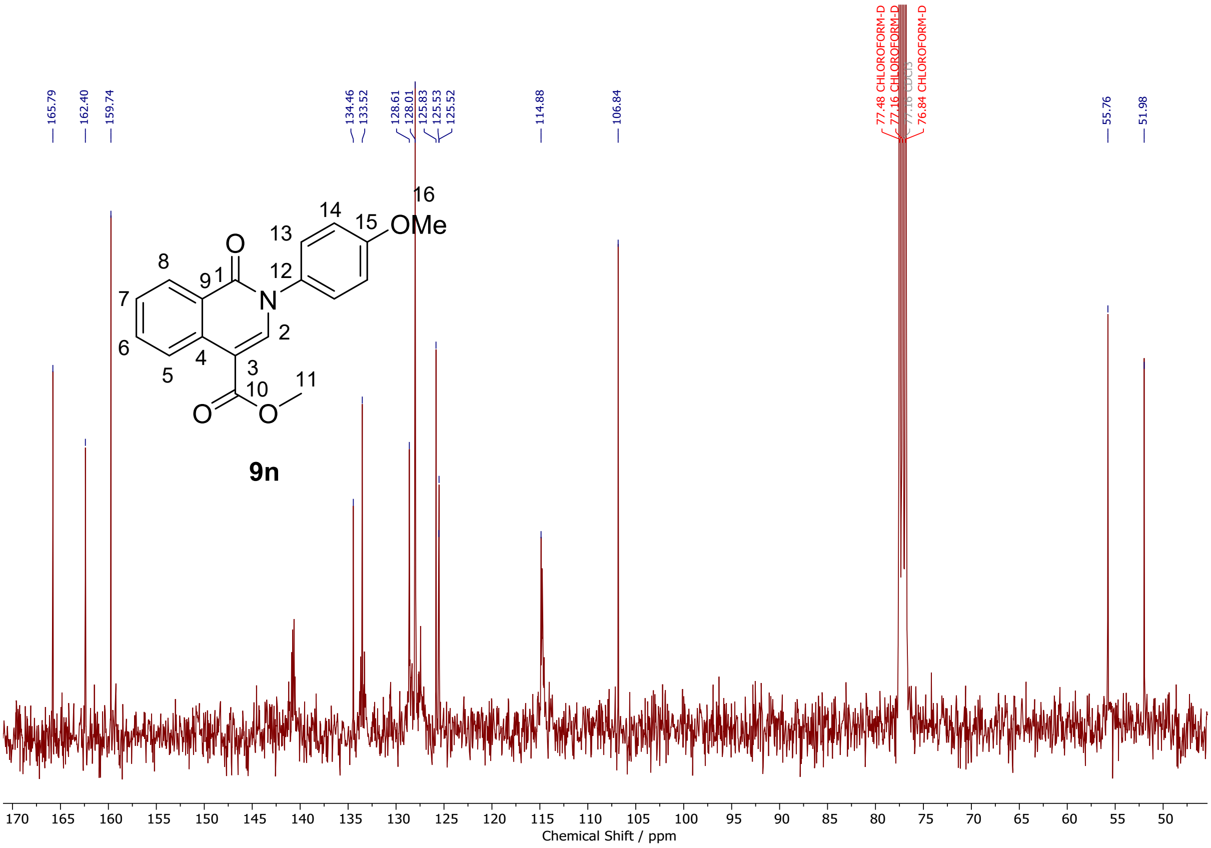

165.79
162.40
159.74
134.46
133.52
128.61
128.01
125.83
125.53
125.52
114.88
106.84
77.48 CHLOROFORM-D
77.16 CHLOROFORM-D
77.16 CDCl3
76.84 CHLOROFORM-D
55.76
51.98
9n
Chemical Shift / ppm

[i] J. Li, B. Qian and H. Huang, *Org. Lett.*, 2018, **20**, 7090–7094.
[ii] L. Yang, M. Liu, Z. Dongkai, H. Xuewen, W. Baolei, S. Zhou and Z. Li, *Chem. Res. Chin. Univ*, 2016, 952–958.
[iii] P.-L. Zhao, C.-L. Liu, W. Huang, Y.-Z. Wang and G.-F. Yang, *Journal of Agricultural and Food Chemistry*, 2007, **55**, 5697–5700
[iv] Q. Wu, G. Wang, S. Huang, L. Lin and G. Yang, *Molecules*, 2010, **15**, 9024–9034.
[v] L. Bao, S. Wang, D. Song, J. Wang, X. Cao and S. Ke, *Molecules*, 2019, **24**, 1304.
[vi] S. D. Mahale, A. Prasad and S. B. Mhaske, *Chem. Commun.*, 2024, **61**, 133–136.
